# Loop dynamics govern MALT1 activation revealed by integrative AlphaFold, MD, and NMR analysis

**DOI:** 10.1038/s41598-026-53505-4

**Published:** 2026-05-20

**Authors:** Dmitry Lesovoy, Tatiana Agback, Konstantin Roshchin, Tatyana Sandalova, Adnane Achour, Xiao Han, Alexander Lomzov, Vladislav Orekhov, Peter Agback

**Affiliations:** 1 Shemyakin-Ovchinnikov Institute of Bioorganic Chemistry RAS, 117997 Moscow, Russia; 2 Department of Molecular Sciences, Swedish University of Agricultural Sciences, PO Box 7015, 750 07 Uppsala, Sweden; 3 Department of Chemistry and Molecular Biology, University of Gothenburg, Box 465, 40530 Gothenburg, Sweden; 4 Science for Life Laboratory, Department of Medicine, Karolinska Institute, 17165 Solna, Sweden; 5 Division of Infectious Diseases, Karolinska University Hospital, 171 76 Stockholm, Sweden; 6 Laboratory of Structural Biology, Institute of Chemical Biology and Fundamental Medicine SB RAS, 630090 Novosibirsk, Russia; 7 Swedish NMR Centre, Science for Life Laboratory, SciLifeLab, University of Gothenburg, Box 465, 40530 Gothenburg, Sweden

**Keywords:** Biochemistry, Biophysics, Computational biology and bioinformatics, Structural biology

## Abstract

Mucosa-associated lymphoid tissue lymphoma translocation protein 1 (MALT1) is a central regulator of immune signalling, yet how its conformational dynamics govern activation remains poorly understood. Here, we integrate NMR relaxation measurements, molecular dynamics simulations, and ensemble modelling to characterise the solution-state behaviour of the catalytic core MALT1(PCASP–Ig3)_339−719_ under different ionic conditions. Under low-salt conditions, all simulations converge to a dominant inactive ensemble characterised by inward rotation of residue W580 and coordinated rearrangements of regulatory loops, indicating that the inactive state is energetically favoured in solution. At intermediate ionic strength, reversible loop motions permit transient access to active-like conformations, whereas high-salt conditions suppress loop dynamics and trap the protein in its functional starting conformational basin. Comparison with experimental NMR relaxation data identifies the low-salt inactive ensemble as the best representation of solution-state dynamics, revealing stable hydrophobic cores and loop-localised conformational plasticity. Together, these results support ionic strength as a key determinant for MALT1 conformational equilibria in solution and suggest how coordinated loop dynamics regulate access to catalytically competent states. This provides a dynamic framework for future structure-based modulation of MALT1 activity.

**Supplementary Information:**

The online version contains supplementary material available at 10.1038/s41598-026-53505-4.

## Introduction

Mucosa-associated lymphoid tissue lymphoma-translocation protein 1 (MALT1), a crucial human paracaspase, forms the CBM complex with CARD11 and BCL10^1–3^, which plays a central role in antigen receptor–induced activation of the NF-κB signalling pathway. MALT1 is essential for the survival, proliferation, and function of B and T cells, and is therefore a key therapeutic target in lymphoma, other cancers, and autoimmune diseases^4–11^. Assembly of the CBM complex activates the cysteine-dependent protease activity of MALT1, enabling cleavage of multiple substrates involved in signalling, adhesion, transcription, and mRNA regulation^2^. Structurally, MALT1 comprises an N-terminal death domain, two immunoglobulin-like domains (Ig1, Ig2), a paracaspase (PCASP) domain containing the catalytic dyad H415/C464^12,13^, a third immunoglobulin-like domain (Ig3), and a C-terminal region of undefined structure^14,15^. Although all MALT1domains contribute to its cellular function, a minimal construct comprising PCASP and Ig3 displays peptidase in vitro activity under activating conditions, comparable to that of full-length MALT1^15^.

Since its identification as a distant caspase homolog^16,17^, biochemical and structural studies have advanced our understanding of MALT1 activation mechanisms^2,8,9,18–20^. The prevailing model proposes that MALT1 activation requires dimerisation of the PCASP domain, followed by release of Ig3-mediated autoinhibition upon substrate binding^21^.

Supporting this view, crystal structures of MALT1(PCASP-Ig3)_339–719_ revealed an autoinhibited monomeric state, with dimerisation inducible by peptide ligands^15,22^. Biochemical analysis further demonstrated that monomeric MALT1 can spontaneously form dimers in solution, reinforcing the importance of dimerisation for catalytic activation^23^. MALT1 activation thus seems to involve a conformational transition driven by regulatory interactions and ligand binding, which together stabilise the active form and enhance catalytic function^14,15^.

Available crystal structures of MALT1 in active states, typically bound to peptide-based inhibitors^14,15,24,25^, and in inactive states bound to allosteric ligands^25–29^, provide structural snapshots that describe ligand-induced rearrangements in loops 2, 3, and 4.

However, most often, enzymes are inherently dynamic systems that populate ensembles of interconverting conformations, and the role of conformational dynamics in the regulation of MALT1 activation has remained insufficiently explored.

Protein loops frequently play central roles in catalysis, substrate recognition, and allosteric regulation, and their evolutionary conservation^30^ underscores their functional importance^31–39^. Understanding loop dynamics is therefore in our opinion critical for elucidating the molecular basis of MALT1 regulation.

We previously reported near-complete NMR backbone and methyl side-chain resonance assignments for the apo form of human MALT1, MALT1(PCASP-Ig3)_339–719_^40,41^, providing a foundation for quantitative analysis of internal dynamics using relaxation measurements and validation of molecular dynamics (MD) simulations. NMR studies demonstrated that this construct is predominantly monomeric under near-physiological conditions and that the PCASP and Ig3 domains exhibit semi-independent motions, that contrast with the compact interdomain interface in crystal structures^42^.

Complementary AlphaFold2 and AlphaFold3 modelling of the apo form of MALT1(PCASP-Ig3)_339–719_ predicted multiple conformational families that differ in PCASP activity state and the orientation of the key residue W580. Although AlphaFold (AF) predictions lack explicit environmental context, they likely reflect evolutionary constraints encoded in structural databases. Notably, crystallographic studies show that the active PCASP conformation is observed predominantly in ligand-bound states, suggesting that functionally relevant conformations may exist in solution and that these populations could be modulated by environmental factors such as kosmotropic salts^12,43,44^.

The influence of physiological electrolytes on MALT1(PCASP-Ig3)_339–719_ activation remains unclear. High-salt NMR experiments can be technically challenging due to the size of constructs and unfavourable relaxation properties, whereas MD simulations provide a complementary approach to probe electrostatic shielding and salt-dependent conformational shifts.

In this study, we integrated NMR relaxation data, MD simulations, and AF-derived models to characterise the conformational dynamics of MALT1(PCASP-Ig3)_339–719_. Here, AF-derived models are treated as structure-informed starting hypotheses rather than predictors of solution-state populations. Our approach allowed us to define a dominant solution-state ensemble under low-salt conditions, evaluate transitions between inactive and active conformations, and assess the impact of increasing ionic strength on MALT1 structure and dynamics. By integrating AF–MD–NMR data we suggest a unified framework for understanding how environmental factors modulate MALT1 dynamics and activation.

## Results

### Molecular dynamics simulations reveal loop-coupled conformational transitions in MALT1(PCASP-Ig3)_339–719_

To investigate how loop dynamics mediate transitions between inactive (PCASP-I) and active-like (PCASP-A) states, we performed extensive MD simulations under different ionic environments and starting structures (Table 1). Simulations were grouped as follows: trajectories 1–4 sampled low-salt conditions (60 mM NaCl) from AlphaFold starting models, trajectories 5–6 used kosmotropic citrate conditions to mimic in vitro activity assay ionic strength, trajectories 7–9 explored high-salt conditions (500 mM NaCl), trajectories 10–11 were initiated from experimentally determined X-ray inactive structures under low-salt conditions, and trajectory 12 tested the polarizable AMOEBA force field at low salt.

This design enabled direct comparison of conformational dynamics across ionic strengths, starting conformations, and force-field treatments. Analysis focused on backbone root-mean-square deviation (RMSD), principal component, (PC) projections, loop rearrangements, and W580 side-chain orientations to identify dominant solution-state ensembles, and transitions between PCASP-I and PCASP-A conformations.

**Table 1:** MD simulation trajectories for MALT1(PCASP–Ig3)_339–719_.

Trajectory	Starting structure	Force field/duration	Ionic environment	Notes
1	I (AF3 inactive, W580 inward)	CHARMM36/CUFIX, 3 µs	Low-salt, 60 mM NaCl	Low-salt ensemble
2	II (AF2 active, W580 inward)	CHARMM36/CUFIX, 6 µs	Low-salt, 60 mM NaCl	Low-salt ensemble
3	III (AF3 inactive, W580 outward)	CHARMM36/CUFIX, 3 µs	Low-salt, 60 mM NaCl	Low-salt ensemble
4	IV (AF2 active, W580 outward)	CHARMM36/CUFIX, 4 µs	Low-salt, 60 mM NaCl	Low-salt ensemble
5	I (AF3 inactive, W580 inward)	CHARMM36/CUFIX, 6 µs	Kosmotropic, 166.7 mM trisodium citrate	Kosmotropic ensemble
6	II (AF2 active, W580 inward)	CHARMM36/CUFIX, 5.6 µs	Kosmotropic, 166.7 mM trisodium citrate	Kosmotropic ensemble
7	I (AF3 inactive, W580 inward)	CHARMM36/CUFIX, 8.7 µs	High-salt, 500 mM NaCl	High-salt ensemble
8	II (AF2 active, W580 inward)	CHARMM36/CUFIX, 3 µs	High-salt, 500 mM NaCl	High-salt ensemble
9	IV (AF2 active, W580 outward)	CHARMM36/CUFIX, 4 µs	High-salt, 500 mM NaCl	High-salt ensemble
10	V (X-ray inactive, PDB 3V55)	CHARMM36/CUFIX, 3 µs	Low-salt, 60 mM NaCl	X-ray starting structure
11	VI (X-ray inactive, PDB 9MKD)	CHARMM36/CUFIX, 3 µs	Low-salt, 60 mM NaCl	X-ray starting structure
12	I (AF3 inactive, W580 inward)	AMOEBA, 3 µs	Low-salt, 60 mM NaCl	Polarizable force field

1. Trajectory grouping:

Trajectories 1–4: low-salt (60 mM NaCl) using CHARMM36/CUFIX, with AF2/AF3 starting structures.

Trajectories 5–6: kosmotropic (166.7 mM trisodium citrate) using CHARMM36/CUFIX.

Trajectories 7–9: high-salt (500 mM NaCl) using CHARMM36/CUFIX.

Trajectories 10–11: low-salt with X-ray inactive structures.

Trajectory 12: low salt with polarizable AMOEBA force field.

2. Starting structures (Supplementary S1.1i and j):

a. I: AF3 inactive, W580 inward.

b. II: AF2 active, W580 inward.

c. III: AF3 inactive, W580 outward.

d. IV: AF2 active, W580 outward.

e. V: X-ray inactive (PDB 3V55), W580 inward.

f. VI: X-ray inactive (PDB 9MKD), W580 outward.

#### Low-salt conditions favour an inactive MALT1 conformational ensemble

To obtain conformation ensembles whose back-calculated relaxation parameters best matched experimental NMR data for MALT1(PCASP-Ig3)_339–719_, measured in 10 mM Tris (pH 7.6), 50 mM NaCl, and 2 mM TCEP, we performed free MD simulations starting from four initial AlphaFold structures (I–IV; Table 1 and Supplementary S1.1i and j). All starting models were based on the sequence used in the NMR study (BMRB accession code 52265).

**Fig. 1:**
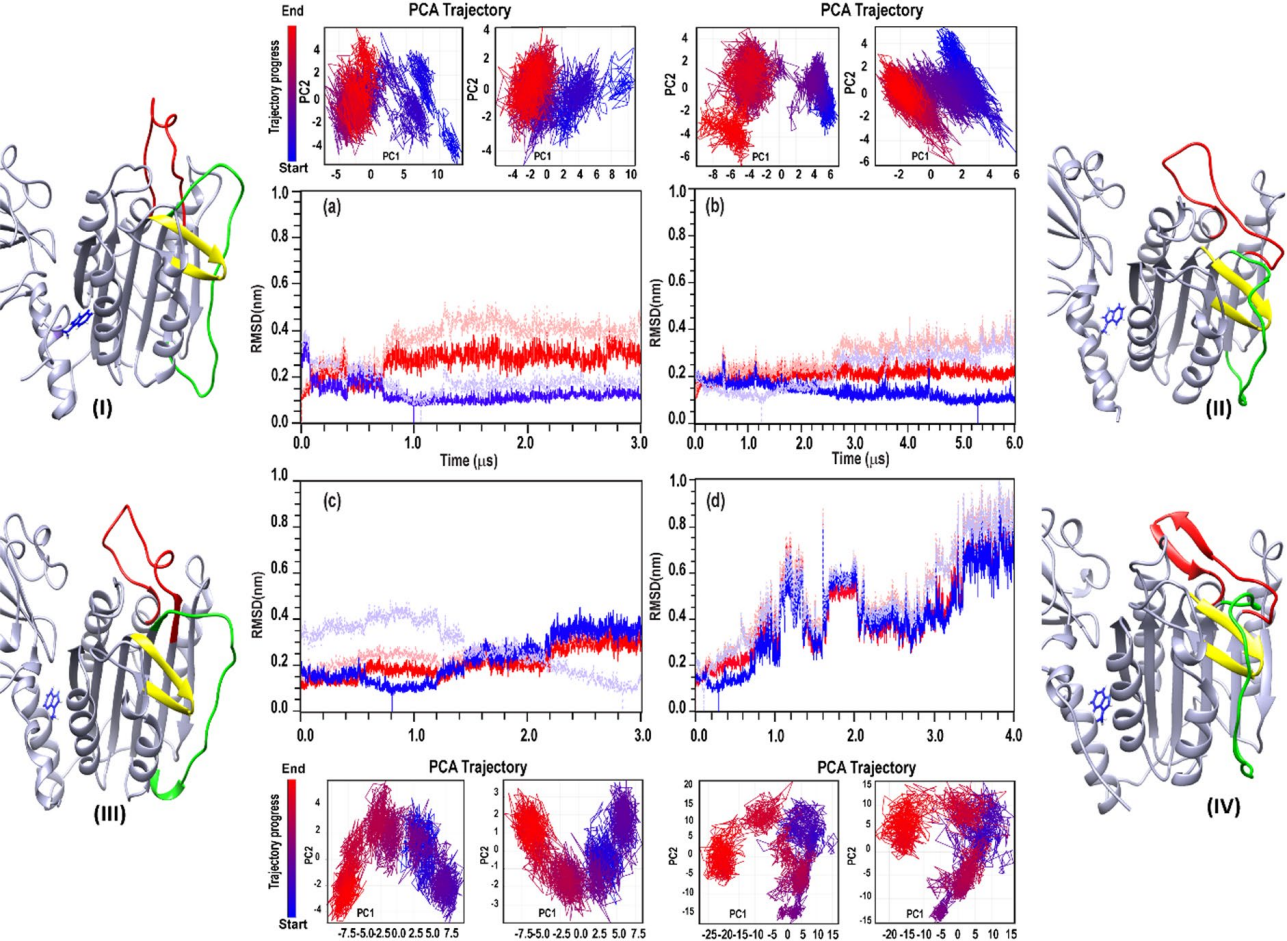
RMSD and PCA of MALT1(PCASP-Ig3)_339–719_ MD trajectories. (**a**–**d**) RMSD of Cα atoms calculated relative to the initial structure (red) or the representative structure of the most populated cluster (blue). RMSD was computed either for selected structured regions, Reg1, (residues 342–467, 484–491, 509–562, and 572–717; dark red/blue) or for all residues, Reg2, excluding the termini (residues 339–341 and 718–725; light red/blue). Trajectories shown are: (**a**) 1, (**b**) 2, (**c**) 3, (**d**) 4. All simulations were performed at 60 mM NaCl. PCA projections (PC1 vs. PC2) are shown above and below each RMSD panel. Left and right PCA panels were calculated using Reg2 and Reg1, respectively. Colours coding highlights the β3 hairpin (residues 416–425), loop 2 (464–485), loop 3 (491–509), and the orientation of W580 (inward or outward, in blue).

The principal component PC1/PC2 projections for individual structures from trajectories 1, 2, 3, and 4 (Fig. 1) show that the sampled conformations do not simply interpolate between two definite states but instead populate a diverse number of ensembles. However, notable differences are observed among trajectories.

The most pronounced changes in RMSD and principal component analysis (PCA) were observed in trajectory 4. This 4 µs MD simulation was initiated from the AF-generated structure IV (Table 1) in which W580 adopts an outward-facing conformation and the PCASP domain begins in an active state. Here, the RMSD profile of trajectory 4 indicates structural instability (Fig. 1d), consistent with an ongoing conformational transition.

**Fig. 2:**
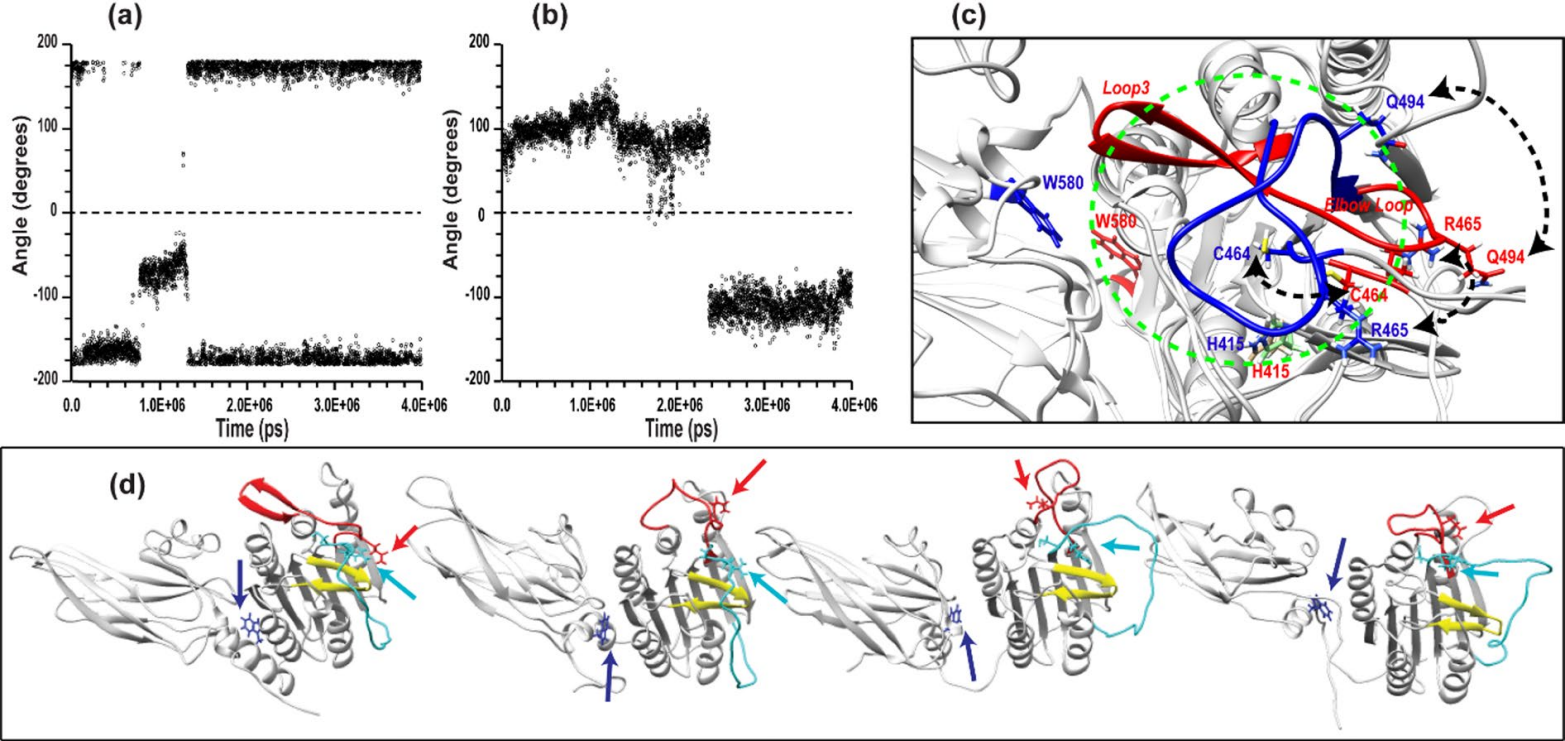
Dynamics of χ_1_ and χ_2_ angles along MD trajectory 4 of MALT1 (PCASP-Ig3)_339–719_. (**a**) χ_1_ and (**b**) χ_2_ torsion angles of W580 monitored throughout the 4 µs MD simulation under low-salt conditions, shown relative to the initial structure IV (Table 1). (**c**) Superimposed structures from the start IV and end of trajectory 4, illustrating the transitions between active and inactive conformations of the PCASP domain. Loop3 and selected residues are highlighted in red and blue, respectively, with the rest of the protein shown in grey. The position of the elbow loop (residue 491–498) in the active conformation is labelled as “Elbow Loop”. Dash lines indicate the displacement of residues C464, R465, and Q494. Two orientations of the W580 aromatic ring and the position of catalytic H415 are shown. The protein’s active site is marked with a dashed green circle. (**d**) Conformational transition of the PCASP domain from active (leftmost structure) to inactive (rightmost structure) state during the 4 µs MD simulation, highlighting the flip of W580. The β3 hairpin (residues 416–425), loop 2 (residues 464–485), and loop3 (residues 491–509) are coloured yellow, cyan, and red, respectively. W580 and Q494 are shown in blue and red, respectively, while R465 and C464 are shown in cyan.

Detailed analysis of this trajectory (described in Supplementary S2.1) reveals a key structural event involving the conformational flip of the aromatic ring of residue W580, associated with transitions in the χ_1_ and χ_2_ dihedral angles (Fig. 2a, b).

Initially, the χ_1_ angle remains stable around − 170° but shifts to − 70° at ~ 800 ns before reverting to − 170° by ~ 1500 ns. During this interval, the χ _2_ angle fluctuates around 100°. At ~ 2300 ns, a pronounced ring flip occurs, as χ_2_ shifts from 80° to − 100°.

Following this event, both angles stabilize, and the system adopts an inward-facing W580 conformation (Fig. 2c, d).

The system, which began in the active conformation, transitions into an inactive state resembling that observed in trajectory 1 (Fig. 2c, d). This transition is driven by coordinated movements of loop 2 and loop 3.

A distinct two-site flip of loop 2 (cyan in Fig. 2d) across the β3 hairpin (residues 416–425, yellow) occurs at approximately 2.5 µs, placing loop 2 in a position similar to that seen in the inactive starting structure I of trajectory 1. This repositioning of loop 2 subsequently displaces loop 3 toward the top of the MALT1 active site (Fig. 2c).

These rearrangements are captured by conformational “fingerprints” involving residues C464, R465, E549, and Q494, previously identified through comparisons of MALT1 crystal structures bound to allosteric or peptide inhibitors. In the inactive state, loop 3 folds over the catalytic dyad C464–H415, effectively blocking substrate access. This transition is accompanied by large positional shifts of Q494 and R465 relative to E549 (Detailed analysis in Supplementary S2.1).

Similar to trajectory 4, trajectory 3, initiated from the outward-facing W580 conformations with the PCASP domain in the inactive state (structure III), shows a strong tendency for the aromatic ring of W580 to flip from an outward- to an inward-facing conformation. This behaviour is reflected in increased RMSD variability although less pronounced than in trajectory 4, and in the conformational diversity observed in the PC1/PC2 projections (Fig. 1c). PCA loadings for trajectory 3 further illustrate that despite tendency to the transition of W580 toward an inward-facing conformation in MALT1(PCASP-Ig3)_339–719_, the positions of loop 2 and loop 3 remain characteristic of the inactive state (Fig. 1c and Figure S1aB) (Detailed analysis in Supplementary S2.2).

In contrast, trajectory 1, initiated from an inward-facing W580 conformation with an inactive PCASP domain (structure I), shows structural stability after an initial equilibration period. The system remains confined to a single dominant conformational cluster throughout the simulation (Fig. 1a and Figure S1aA) and (Supplementary S2.3).

Finally, we investigated whether a spontaneous transition from the active PCASP conformation to the inactive, inward-facing, W580 conformation could be captured in longer MD simulations under low-salt conditions (60 mM NaCl).

To address this, we performed an additional 6 µs MD simulation (trajectory 2), starting from the AF-generated structure (Table 1) with the PCASP domain in the active conformation. This stands in contrast to trajectory 1, which begins with the PCASP domain in the inactive state, although both simulations were based on an inward-facing W580 conformation. RMSD and PCA analysis reveal a transition at ~ 2.5 µs (Fig. 1b, light pink and grey curves) driven by the flip of loop 2 around the β-sheet spanning residues 416–426, leading to conversion from the active to the inactive PCASP conformation (Supplementary S2.4, Figure S1aC).

In summary, all four trajectories, despite distinct starting active-site conformations and W580 orientations, converged under low-salt conditions (60 mM NaCl) to a common ensemble characterized by an inward-facing W580 aromatic ring and an inactive PCASP active site in MALT1(PCASP-Ig3)_339–719_.

#### Dynamics of MALT1(PCASP–Ig3)_339−719_ under kosmotropic conditions

To examine how elevated ionic strength influences the conformational dynamics of MALT1(PCASP–Ig3)_339-719_, MD simulations were performed under kosmotropic conditions using trisodium citrate at concentrations corresponding to those employed in in vitro activity assays (166.7 mM). These conditions correspond to a high–ionic-strength environment in which electrostatic shielding shifts the balance between electrostatic and hydrophobic interactions within the protein.

Simulations were initiated from two starting structures, I and II, both featuring inward-facing W580 conformations with either inactive or active-like PCASP domains creating trajectories 5 and 6, respectively (Table 1).

In simulations initiated from the inactive PCASP conformation (trajectory 5), the system remained structurally stable throughout the simulation, with RMSD and PCA profiles closely resembling those observed under low-salt conditions for the inactive ensemble of trajectory 1 (Figure S1cA and Figure S2j), indicating persistence of the inactive conformational basin.

**Fig. 3:**
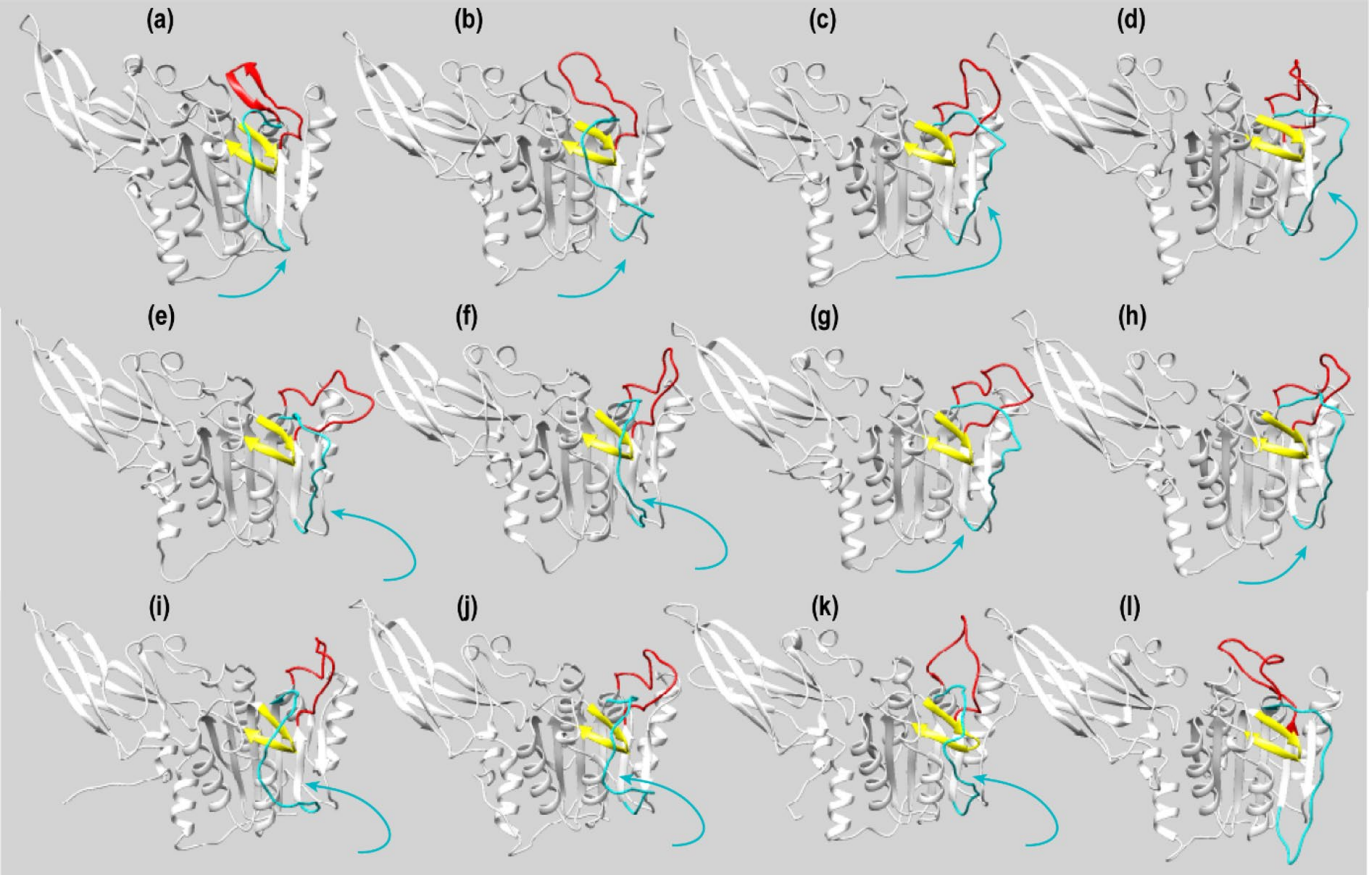
Structural dynamics along MD Trajectory 6 of MALT1(PCASP–Ig3)_339−719_ under kosmotropic conditions. (**a**–**k**) Selected snapshots from MD trajectory 6 performed at 166.7 mM trisodium citrate illustrating the conformational transition of loop 2 (residues 464–485, cyan) around the β3 hairpin (residues 416–425, yellow) in MALT1(PCASP–Ig3)_339−719_. The starting structure (**a**) corresponds to the active conformation of the PCASP domain. Structures (**b**–**k**) were extracted at 300, 1710, 1740, 2220, 2670, 3060, 3120, 3150, 3210, and 3840 ns, respectively. (**l**) The inactive PCASP-domain conformation modelled on the crystal structure PDB ID: 3V55.

In contrast, simulations initiated from an active-like PCASP conformation, (structure II, trajectory 6) revealed qualitatively distinct behaviour. Rather than converging to a single inactive ensemble, the PCASP domain sampled reversible transitions between active-like and inactive-like loop 2 configurations within the accessible MD timescale (Fig. 3b–d). These transitions were primarily mediated by the repeated repositioning of loop 2 across the β3 hairpin and were accompanied by correlated fluctuations of loop 3 (Fig. 3 and S1cC).

Notably, although loop 2 exchanged between active- and inactive-like positions, the system did not irreversibly collapse into the inactive ensemble observed under low-salt conditions. Instead, the trajectory populated multiple conformational basins, suggesting that kosmotropic ionic conditions partially stabilise active-like states while preserving access to inactive-like configurations (Fig. 3e).

Such reversible loop rearrangements were not observed in simulations performed either under low-salt conditions (trajectory 1) or in kosmotropic simulations initiated from inactive PCASP conformations (trajectory 5), even over extended simulation times. These observations indicate that elevated ionic strength reshapes the free-energy landscape governing loop motions, lowering the barrier for interconversion between active- and inactive-like loop arrangements without enforcing a single dominant state.

Together, these results suggest that under kosmotropic conditions relevant to in vitro activity assays, MALT1(PCASP–Ig3)_339−719_ exhibits enhanced loop plasticity and reversible sampling of active-like conformations. This behaviour contrasts with the strong bias toward inactive states observed at low ionic strength.

#### Dynamics of MALT1(PCASP–Ig3)_339-719_ under high-salt conditions (500 mM NaCl)

To assess the effect of maximal electrostatic shielding on MALT1 conformational dynamics, MD simulations were performed at high ionic strength (500 mM NaCl). Under these conditions, long-range electrostatic interactions are strongly attenuated, providing a stringent test of the role of electrostatics in the regulation of loop mobility, and active-site accessibility.

Unlike kosmotropic citrate conditions, high NaCl primarily increases ionic strength without introducing strong Hofmeister-specific effects^43–45^.

Simulations were initiated from three starting conformations (I, II, and IV), differing in W580 orientation and PCASP activity state generating trajectories 7–9, respectively (Table 1).

In simulations initiated from inactive PCASP conformations with inward-facing W580, (structure I; trajectory 7), the protein remained structurally stable throughout the simulation. RMSD and PCA profiles indicating confinement to a single conformational basin, with no evidence of large-scale loop rearrangements (Figure S2g and S1bA).

**Fig. 4:**
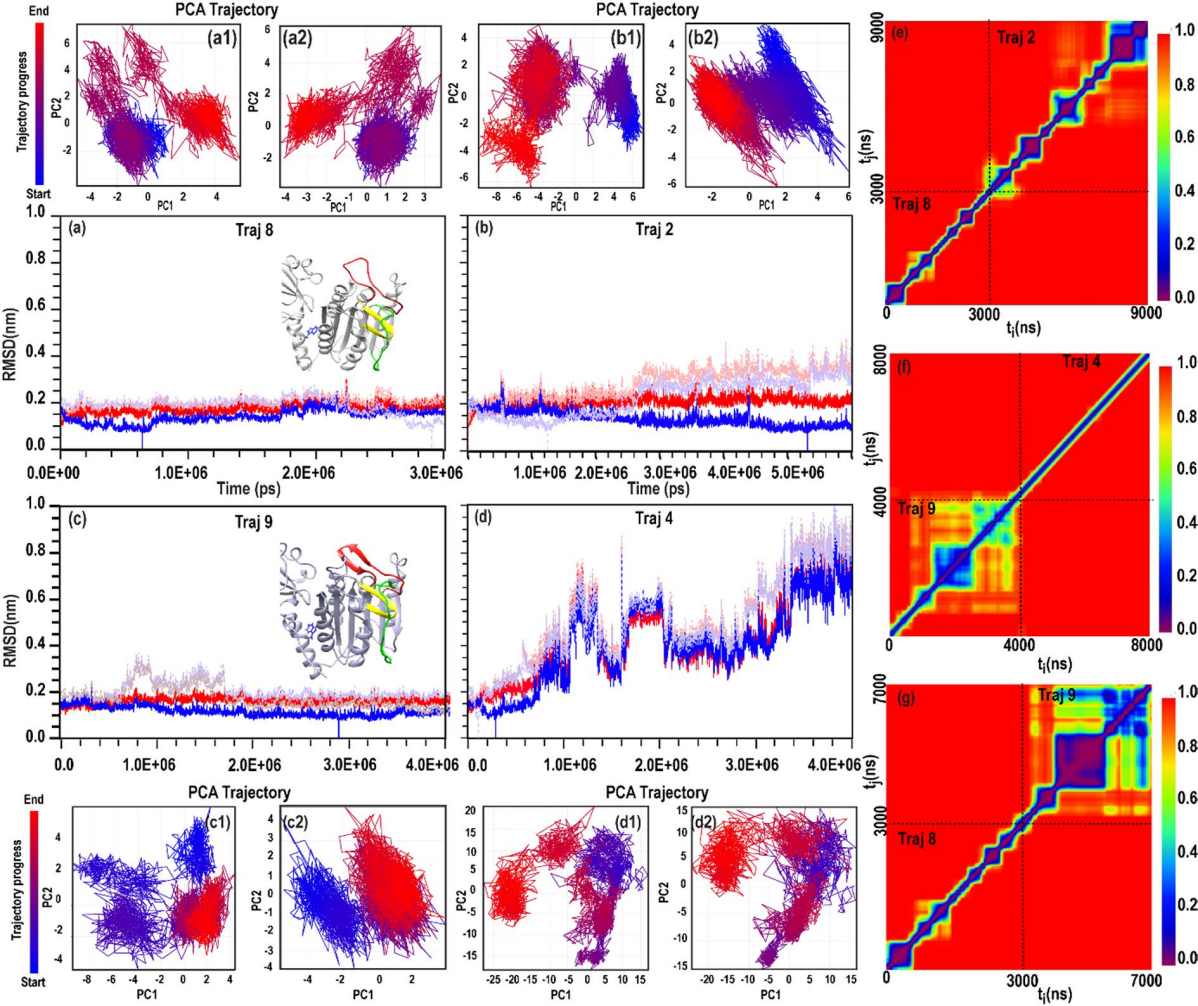
Conformational dynamics of MALT1(PCASP-Ig3)_339-719_ under high ionic strength (500 mM NaCl). (**a**–**d**) Backbone RMSD of MD trajectories 8 (**a**), 2 (**b**), 9 (**c**), and 4 (**d**), calculated relative to the respective starting structures (red) and to the representative structure of the most populated cluster (blue). PC1 vs. PC2 projections of trajectories 8 (**a1**,**a2**), 2 (**b1**,**b2**), 9 (**c1**,**c2**) and 4 (**d1**–**d2**) illustrate the distribution of conformational states following equilibration. Panels (**e**–**g**) show the conformational clustering of the combined trajectories 8 and 2, 9 and 4, 9 and 8, respectively.

Similarly, simulations initiated from an active PCASP conformation with outward-facing W580, (structure IV; trajectory 9) showed pronounced structural stability. In contrast to behaviour observed under low-salt conditions, no spontaneous transition toward the inactive ensemble was detected (Fig. 4c, d). W580 retained its outward orientation, loop fluctuations were strongly dampened, and the active-site architecture remained intact over the full simulation timescale (Figure S1bD).

PCA and conformational clustering further revealed that high-salt simulations populate well-defined, non-overlapping conformational minima. This behaviour contrasts sharply with the broad and interconverting ensembles observed at lower ionic strength (Fig. 4c, d) and indicates that elevated ionic strength suppresses large-scale loop rearrangements and restricts conformational exploration.

We next performed a 3 µs simulation (trajectory 8) starting from the same AF-generated structure used for trajectory 2, in which W580 is inward-facing and the PCASP domain adopts an active conformation. RMSD profiles for trajectories 8 and 2 are similar and, taken alone, less conclusive.

However, RMSD analysis of trajectory 8 (Fig. 4a) indicates a stable structure with no major deviations from the starting conformation. Structural inspection confirms that W580 remains inward-facing and that the active site architecture of the PCASP domain is preserved throughout the simulation (Supplementary S2.5; Figure S1bC).

To directly compare salt-dependent effects, combined clustering analysis were performed on trajectories initiated from identical starting structures but simulated under different ionic strengths (see Methods 4.4).

As shown in Fig. 4f, where trajectories 9 and 4 are combined, whereas the low-salt trajectories 4 rapidly dispersed across conformational space, the corresponding high-salt trajectories 9 remained confined to narrow minima, indicating a strong stabilising effect of ionic shielding on the starting conformations.

Clustering of the combined trajectories 8 and 2 (Fig. 4e) reveals several local minima, indicating conformational heterogeneity despite the absence of large-scale transitions.

Notably, trajectory 9 is more structurally stable than trajectory 8 under high-salt conditions, as demonstrated by clustering of the combined trajectories (Fig. 4g).

Trajectory 9 occupies a single, well-populated local minimum, whereas trajectory 8 populates several smaller, less stable clusters, with no overlap between their respective distributions.

Across all high-salt simulations, neither active-to-inactive nor inactive-to-active transitions of the PCASP domain were observed within the accessible MD timescale. This behaviour contrasts with the reversible loop rearrangements detected under intermediate ionic strength and with the strong bias toward inactive states observed at low salt.

Together, these results suggest that high ionic strength effectively quenches the loop dynamics required for interconversion between active and inactive states. As a consequence, MALT1(PCASP–Ig3)_339-719_ becomes trapped within a conformational basin defined by the starting state.

#### Comparison of MD ensembles with experimental backbone relaxation

To assess which MD-derived ensembles best represent the solution-state dynamics of MALT1(PCASP–Ig3)_339−719_, backbone^15^ N relaxation parameters (R_1_, R_2_, ^1^-^15^ N NOE) and CSA/dipole cross-correlated relaxation rates (η_xy_) were back-calculated from selected MD trajectories (Supplementary S3.4.2) and compared with experimental NMR data acquired under low-salt conditions.

**Fig. 5:**
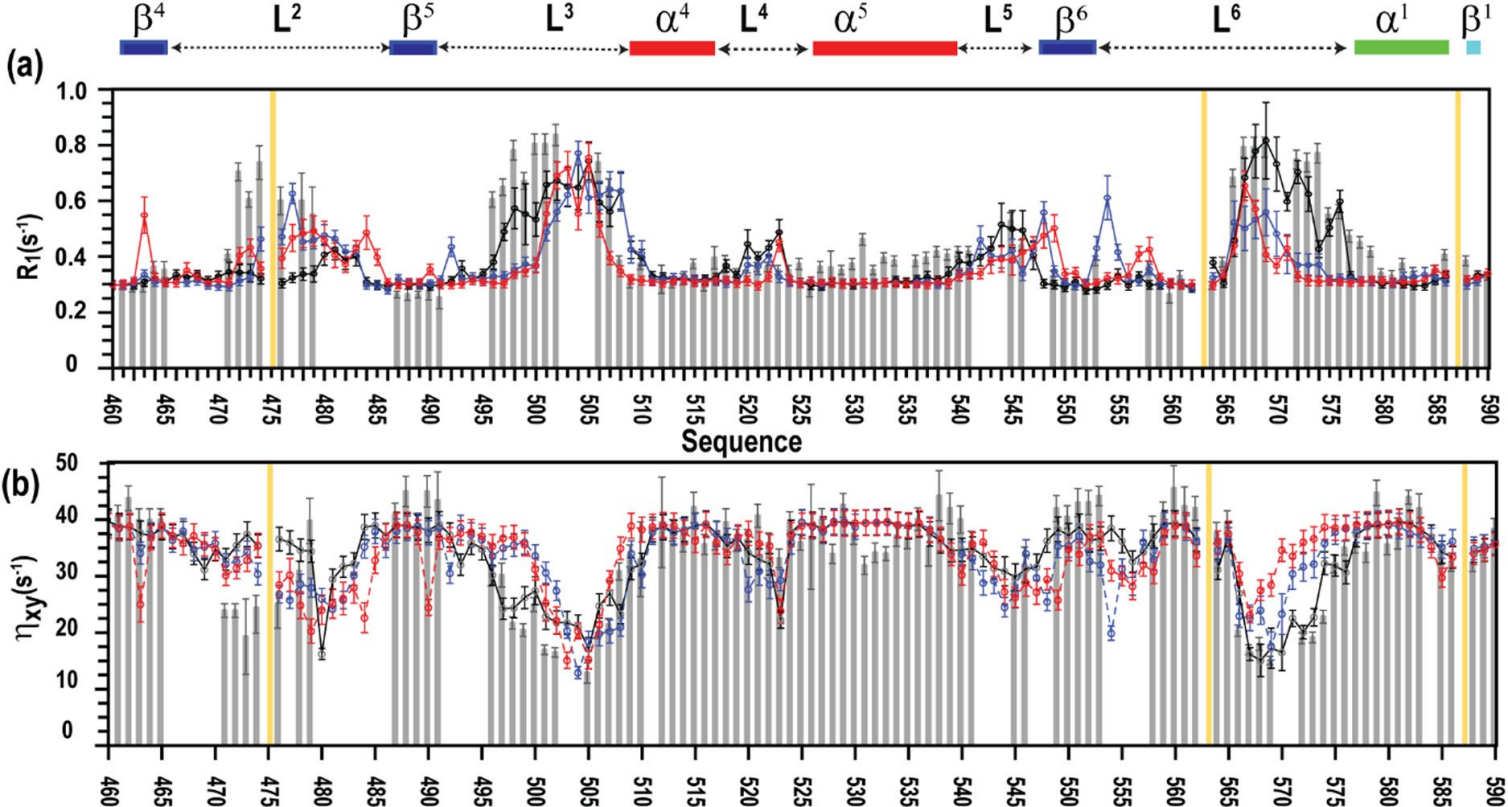
Backbone amide relaxation parameters of MALT1(PCASP-Ig3)_339–719_, measured at 900 MHz. Backbone amide relaxation parameters for residues 460–590 of MALT1(PCASP-Ig3)_339–719_ are shown: (**a**) longitudinal relaxation rate (R_1_, s^− 1^), and (**b**) ^1^-^15^ N CSA/dipole cross-correlation relaxation rates, η_xy_. Experimentally measured R_1_ and η_xy_ values are shown as light grey solid bars. Corresponding parameters back-calculated from molecular dynamics ensembles 8, 9, and 1 are shown as solid blue, red and black lines, respectively. Ensembles 8 and 9 were generated under high salt conditions from trajectory segments 2500–3000 ns and 3500–4000 ns, respectively, using the CHARMM36/CUFIX force field. Ensemble 1 was generated under low salt conditions from trajectory segments 2500–3000 ns using the same force field. Experimental error bars represent one standard deviation from curve fitting; uncertainties in back-calculated parameters were estimated by bootstrap analysis.

Analysis focused on representative trajectories corresponding to distinct conformational states: an inactive ensemble with inward-facing W580 (trajectory 1) and active-like ensembles with W580 oriented inward or outward (trajectories 8 and 9, respectively). For each trajectory, relaxation parameters were calculated from equilibrated MD segments and compared with experiment (Fig. 5; Supplementary Figure S3).

Across the structured core of the protein, calculated relaxation parameters showed good overall agreement with experiment for all ensembles (Supplementary Figure S3).

The largest discrepancies were confined to flexible loop regions, particularly loop 3 (residues 496–510) and loop 6 (residues 566–580). This may reflect contributions from slow conformational exchange processes that are not explicitly captured by back-calculation methods.

To quantify agreement between experimental and calculated R_1_, R_2_, ^1^-^15^ N NOE and η_xy_ values, two complementary scoring approaches were applied. First, a cosine-based criterion (Eq. 1 in Method 4.4) was used to rank trajectories according to overall similarity. Second, RMSE and MAE values (Supplementary S3.5) were computed to the best-scoring trajectory. Complete results from the cosine ranking and RMSE analysis for all trajectories are presented in Table S2.

Using the cosine-based metric, trajectory 1 achieved the lowest overall score and the closest agreement with experimental R_1_, R_2_, ^1^-^15^ N NOE and η_xy_ values, when compared with active-like ensembles (trajectories 8 and 9).

Quantitative comparison across multiple scoring metrics further identified the inactive ensemble sampled in trajectory 1 as providing the most consistent agreement with experimental relaxation data across all parameters (Table S2).

In particular, the agreement in η_xy_ values, which are insensitive to slow exchange contributions, supports the conclusion that the inactive ensemble sampled under low-salt conditions in trajectory 1 most faithfully represents the dominant solution-state conformations of MALT1(PCASP–Ig3)_339−719_.

Together, these results validate the MD ensemble initiated from the NMR-consistent inactive structure as an accurate representation of backbone dynamics and establish a benchmark MD-protocol for interpreting salt-dependent conformational changes.

#### Comparison of MD ensembles initiated from AlphaFold and X-ray structures

To evaluate the dependence of MD-derived conformational ensembles on the choice of starting structures, we compared simulations initiated from AlphaFold/NMR-consistent models with those initiated from experimentally determined inactive X-ray structures of MALT1(PCASP–Ig3)_339-719_.

**Fig. 6:**
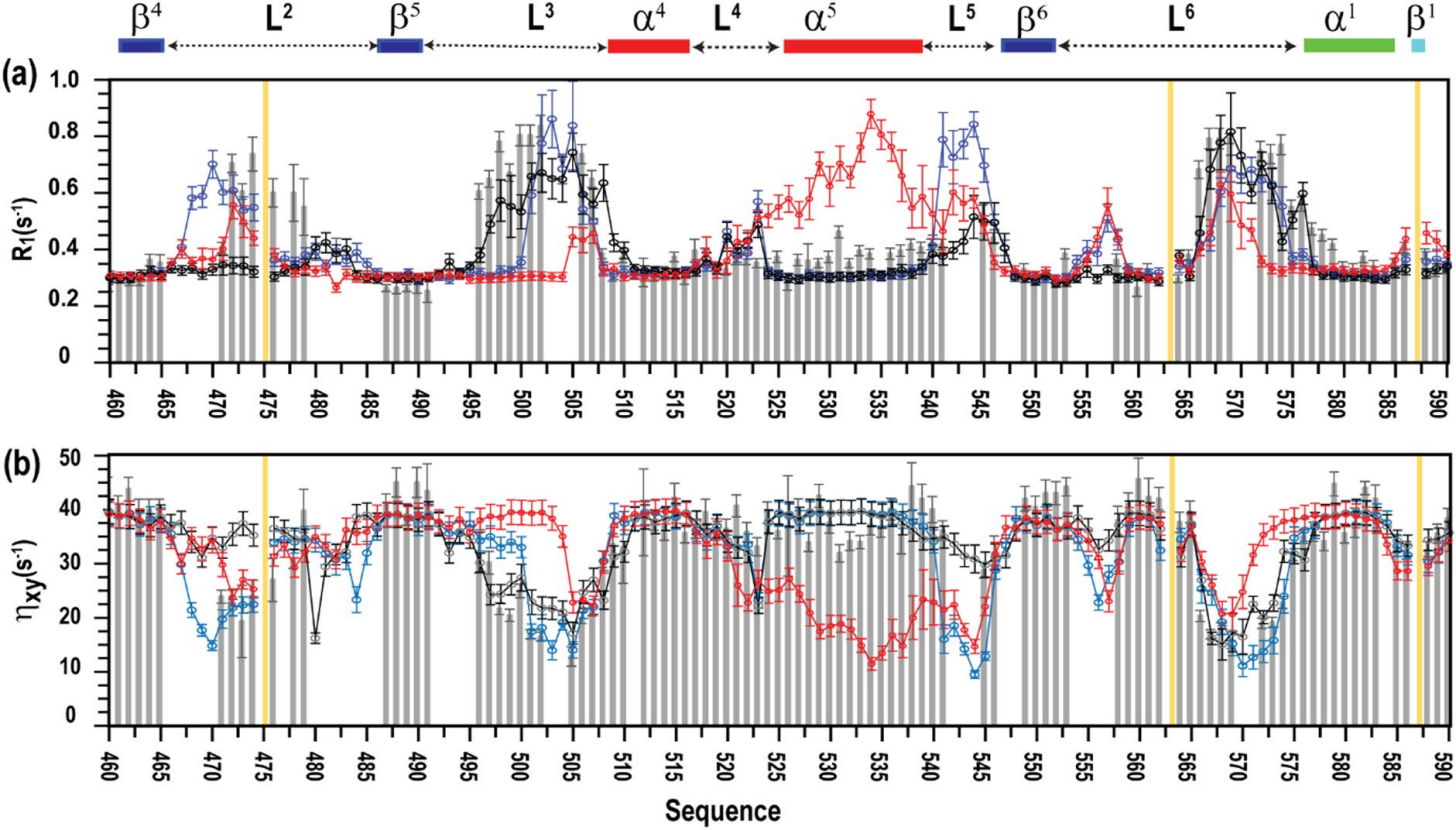
Backbone amide relaxation parameters of MALT1(PCASP-Ig3)_339–719_ measured at 900 MHz. Backbone amide relaxation parameters for residues 460–590 of the MALT1(PCASP-Ig3)_339–719_ are shown: (**a**) longitudinal relaxation rate (R_1_, s^− 1^), and (**b**) ^1^-^15^ N CSA/dipole cross-correlation relaxation rates, η_xy_. Experimentally measured R_1_ and η_xy_ values are shown as light grey bars. Corresponding parameters back-calculated from molecular dynamics ensembles 10, 11 and 1 are shown as solid blue, red and black lines, respectively. Ensembles 10, 11 and 1 were generated from trajectory segments spanning 2500–3000 ns using the CHARMM36/CUFIX force field. Experimental error bars represent one standard deviation from curve fitting; uncertainties in back-calculated parameters were estimated by bootstrap analysis.

Three trajectories simulated under identical low-salt buffer conditions were analysed: trajectory 1, initiated from the AF3-derived structure (structure I); trajectory 10, initiated from the canonical inactive X-ray structure (V) (PDB ID: 3V55); and trajectory 11, initiated from a recently determined inactive X-ray structure (structure VI; PDB ID: 9MKD) exhibiting altered loop 3 and α5 conformations (Supplementary Figure S9). All trajectories were analysed using identical MD protocols and backbone relaxation back-calculation procedures (Fig. 6; Supplementary Figure S4).

Comparison of backbone relaxation parameters revealed that trajectories 10 and 1 produce highly similar dynamic profiles, with only minor differences localised to loop 2. In contrast, trajectory 11 showed pronounced deviations from experimental relaxation data, particularly in regions corresponding to the α5 helix and adjacent loops segments (Fig. 6; Supplementary Figure S4).

Quantitative scoring confirmed that ensembles derived from the AF3-consistent model and from the canonical inactive X-ray structure (3V55) reproduce the experimentally observed backbone dynamics with comparable accuracy. By contrast, the ensemble derived from the alternative inactive X-ray structure (9MKD) exhibits substantially poorer agreement with experimental data (Table S2).

These results demonstrate that MD simulations initiated from structurally consistent inactive conformations converge toward similar solution-state ensembles, independent of whether the starting model is derived from AlphaFold or crystallography. In contrast, starting structures with atypical loop or secondary-structure features can bias the resulting ensemble away from experimentally observed dynamics.

### Methyl group dynamics reveal stability of hydrophobic cores

To assess whether large-scale conformational transitions of MALT1 affect fast side-chain dynamics, we compared experimentally measured methyl relaxation parameters with values back-calculated from MD simulations performed under low- and high-salt conditions.

Back-calculated ^13^C-^1^H methyl longitudinal (R_1_) and cross-correlated (Γ_2_) relaxation rates from representative inactive (trajectory 1) and active-like (trajectories 8, 9) ensembles show close agreement with experimental data across Ile, Leu, and Val residues (Supplementary S2.6 Figure S5). This agreement indicates that the MD simulations reliably capture fast side-chain motions, largely independent of the global conformational state.

Analysis of methyl relaxation parameters reveals that assigned methyl groups cluster into four hydrophobic regions (Cl1–Cl4) distributed across the Ig3 and PCASP domains (Supplementary S2.7, Figure S6).

Clusters located within the Ig3 domain (Cl1) and within the PCASP β-sheet core (Cl3 and Cl4) display nearly identical relaxation behaviour across inactive and active-like ensembles, indicating that these regions remain dynamically stable despite pronounced loop rearrangements.

Notably, the interdomain cluster at the Ig3–PCASP interface (Cl2), previously implicated in allosteric ligand binding, also exhibits highly similar methyl relaxation profiles across all ensembles examined (Supplementary S2.7 Figure S6). This observation persists despite substantial differences in active-site configuration and loop positioning between inactive and active-like states.

Together, these results suggest that hydrophobic clusters within MALT1(PCASP–Ig3)_339-719_ act as rigid structural anchors that preserve fast side-chain dynamics across conformational states. Functional transitions of the PCASP domain therefore arise primarily from coordinated loop rearrangements rather than from changes in the dynamics of the hydrophobic core.

### Backbone dynamics localise conformational plasticity to loop regions

To further characterise residue-level dynamics within the MALT1(PCASP–Ig3)_339-719_ ensembles, backbone flexibility was analysed using Cα root-mean-square fluctuations (RMSF) and NH-vector autocorrelation functions derived from MD simulations.

**Fig. 7:**
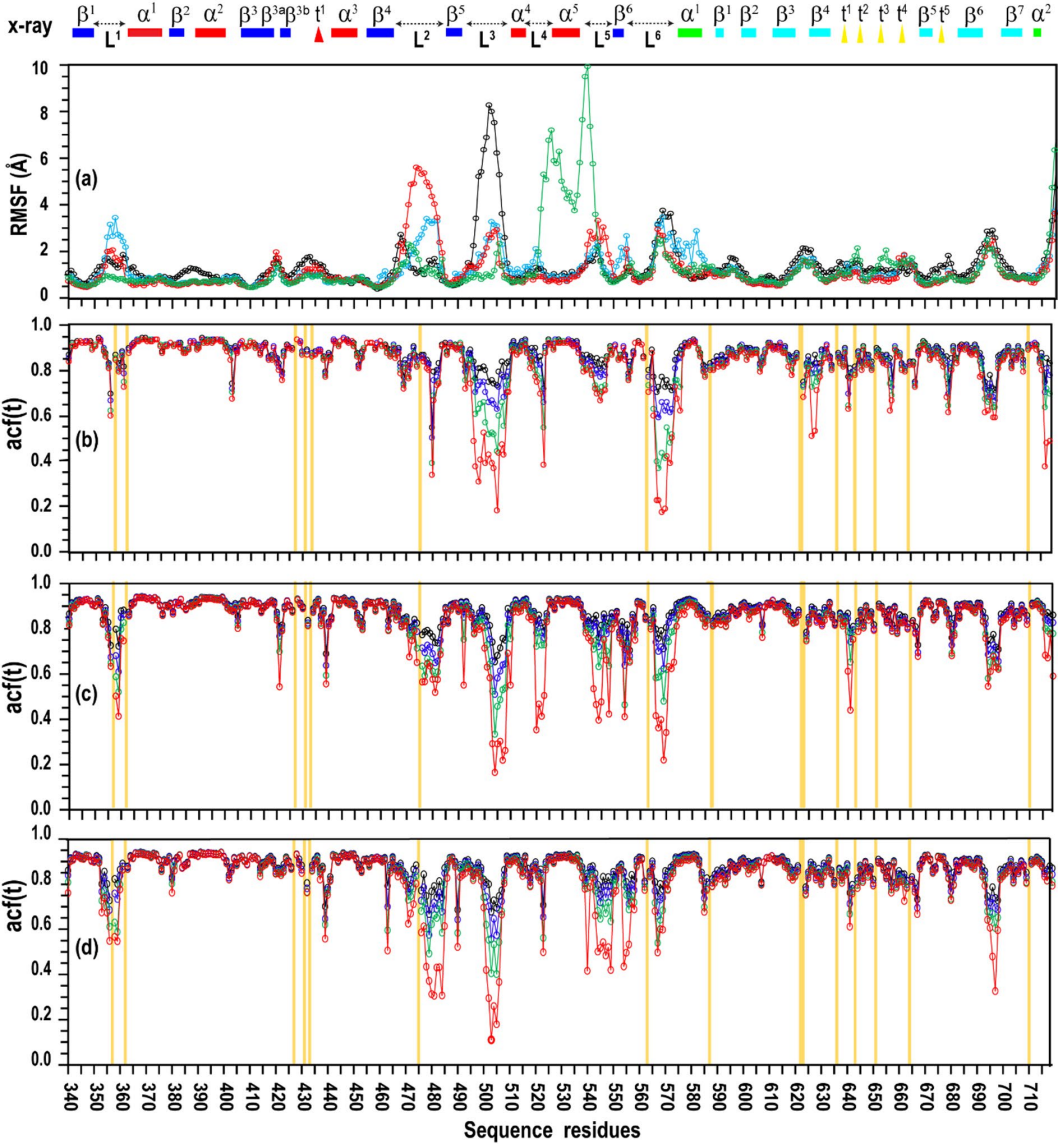
Backbone dynamics of MALT1(PCASP–Ig3)_339-719_ MD ensembles. (**a**) Average backbone root-mean-square fluctuations (RMSF) for ensembles derived from trajectories 1 (black), trajectory 8 (blue), trajectory 9 (red), and trajectory 11 (green) are represented in a line chart. (**b**–**d**) Residue-resolved NH bond vector autocorrelation function values, acf(t), evaluated at t = 0.1, 1.0, 10, and 100 ns (black, blue, green, and red curves, respectively), calculated from MD simulations of MALT1(PCASP–Ig3)_339-719_. Data are shown for trajectories 1 (**b**), 8 (**c**) and 9 (**d**). Secondary structure elements from the crystal structure (PDB 3V55) are indicated above the plots: in the PCASP domain, β-strands (dark blue boxes), α-helices (red boxes), and loops (dashed arrows); in the Ig3 domain, β-strands (light blue boxes), α-helices (green boxes), and short turns (yellow triangles). Proline residues in the protein sequence are indicated by yellow boxes in panels (**b**–**d**).

RMSF profiles for trajectories 1, 8, 9 and 11 reveal that the structured core of the protein remains highly stable across all ensembles, with fluctuations generally below ~ 2.5 Å (Fig. 7a). In contrast, pronounced flexibility is localised to loop regions, particularly loop 2, loop 3, and the PCASP–Ig3 linker (loop 6), consistent with their proposed roles in mediating conformational transitions.

Distinct loop-specific patterns emerge between inactive and active-like ensembles. In the inactive ensemble (trajectory 1), loop 3 exhibits the largest fluctuations, whereas loop 2 remains comparatively ordered. In active-like ensembles (trajectory 8, 9), loop 2 displays increased flexibility, consistent with conformational strain preceding and transition toward the inactive state. Other loops, including loop 1 and loop 5, remain comparatively rigid across all conditions examined.

This contrasts with the RMSF values obtained for conformational ensemble trajectory 11, in which increased flexibility is observed in the α5 helix and loop 5 regions (RMSF ≈ 8–10 Å), indicating substantial disordering of this region of MALT1.

Complementary analysis of NH-vector autocorrelation functions reveals pronounced time-dependent decay in loop regions, indicative of slow backbone motions extending into the hundreds of nanoseconds timescale (Fig. 7b–d). In contrast, structured regions maintain high autocorrelation values across all timescales analysed, reflecting predominantly fast picosecond motions (see Supplementary S2.8 for details).

Together, RMSF and autocorrelation analysis suggest that conformational plasticity in MALT1(PCASP–Ig3)_339-719_ is concentrated within a defined subset of regulatory loops, whereas the domain cores remain dynamically stable. These findings reinforce the conclusion that loop dynamics, rather than large-scale domain rearrangements or destabilisation of the hydrophobic core, govern transitions between inactive and active-like states.

## Discussion

MALT1 is a large multidomain protein whose biological activity is associated with coordinated interdomain motions. Even the truncated construct studied here, MALT1(PCASP-Ig3)_339–719_, retains a complex architecture in which the Ig3 and PCASP domains form an interconnected framework of α-helices, β-strands, linkers, and loops. Although this interdomain arrangement appears static in available crystal structures, our previous work suggested semi-independent motions of the PCASP and Ig3 domains pivoting around W580, suggesting that interdomain flexibility is an intrinsic feature of MALT1 regulation.

While the present analysis focuses on the minimal PCASP–Ig3 construct, this region constitutes the catalytic core of MALT1 and recapitulates key regulatory features observed in the full-length protein. The proposed mechanism therefore reflects intrinsic dynamics of this catalytic core and should not be directly extrapolated to the full-length protein. Interactions mediated by the N-terminal domains and CBM complex assembly are expected to further modulate these transitions in vivo, rather than replace, the intrinsic loop-coupled dynamics described here.

In the present study, we focused on the role of loop dynamics in mediating transitions between inactive and active-like conformations of MALT1(PCASP-Ig3)_339–719_.

NMR relaxation measurements revealed pronounced loop-specific motional differences, with loop 3 displaying substantially higher flexibility than loop 1, motivating a detailed analysis of loop-mediated conformational transitions. By integrating experimental NMR data with molecular dynamics simulations and AF-derived models, we examined how loop motions couple to domain-level rearrangements and how these processes are modulated by ionic conditions.

Although AF is designed to predict static protein structures, AF2 and AF3 models of MALT1(PCASP–Ig3)_339–719_ consistently captured multiple conformational families that differ in PCASP activity state and in the orientation of W580. However, these predictions do not directly reflect environmental effects such as ionic strength, which are known to influence enzyme activity and conformational equilibria. Given that MALT1 protease activity is sensitive to salt concentration, and that our NMR experiments were conducted under sub-physiological ionic strength to preserve conformational heterogeneity, we systematically examined how loop dynamics respond to different salt conditions.

In this context, AF-derived models were treated as structure-informed starting hypotheses rather than predictors of solution-state populations, enabling unbiased exploration of conformational transitions by MD.

Under low-salt conditions, all simulations converged to a common inactive-like ensemble characterised by inward-facing W580, regardless of their starting conformations. This behaviour indicates that reduced ionic strength strongly favours the inactive state of MALT1(PCASP–Ig3)_339−719_. A central feature of this transition is the coordinated rearrangement of loop 2 and loop 3, consistent with broader principles of loop interdependence in enzyme regulation. Although loop 2 is poorly resolved in many inactive apo crystal structures, reflecting intrinsic flexibility, it adopts a well-defined conformation in active, inhibitor-bound states, highlighting its functional importance.

Our simulations capture a concerted transition in which loop 2, loop 3, and W580 rearrange cooperatively as the protein relaxes into its inactive conformation. In this state, loop 2 repositions near the β3a–β3b hairpin and β6 strand, stabilising a misaligned catalytic site and sterically blocking the path required for loop 3 to adopt an active-like configuration. Loop 3, which displays substantial conformational heterogeneity in inactive crystal structures, consistently covers the catalytic cleft in the inactive ensemble, thereby preventing substrate access. These observations are consistent with a sequential activation mechanism in which displacement of loop 2 is a prerequisite for loop 3 retraction and opening of the active site.

Although alternative pathways cannot be excluded, this sequence represents the dominant transition observed within the MD-accessible timescale and across independent trajectories.

Within the timescale and resolution accessible to the present simulations, the observed loop rearrangements define a dominant, intrinsic pathway of the PCASP–Ig3 core whose energetic balance and population are expected to be further shaped by CBM complex assembly and cellular context, rather than fundamentally altered.

Importantly, loop 3 alone cannot access a productive conformation without prior rearrangement of loop 2, indicating that MALT1 activation is governed by a coordinated loop network rather than independent local motions. This mechanism explains the stability of inactive conformations even in the absence of allosteric inhibitors and highlights the role of loop coupling in maintaining catalytic suppression.

Simulations performed under higher physiological ionic strength revealed qualitatively different behaviour. At intermediate salt concentrations, reversible exchange of loop 2 between inactive- and active-like positions was observed, accompanied by correlated fluctuations in loop 3. Such behaviour was absent at low salt and suggests that ionic strength modulates the free-energy landscape governing the loop rearrangements. Because NaCl and citrate differ in both ionic strength and ion type, some of the observed effects may reflect ion-specific contributions in addition to general electrostatic shielding.

In contrast, simulations conducted at high ionic strength, as well as under strongly kosmotropic conditions required for in vitro activity assays, showed pronounced stabilisation of the starting conformations. Under these conditions, W580 remained locked in its initial orientation, loop fluctuations were strongly dampened, and no active–inactive transitions were observed.

These findings indicate that elevated ionic strength suppresses the loop dynamics required for conformational transitions, effectively trapping the protein in a single conformational basin. Although quantitative agreement with biochemical measurements may be influenced by current force-field limitations and by the distinction between conformational accessibility and catalytic turnover, the simulations consistently demonstrate that ionic conditions exert a strong influence on the dynamic ensemble of active-like states.

Comparison of MD-derived ensembles with experimental NMR relaxation data allowed us to identify trajectories that best represent the solution-state behaviour of MALT1(PCASP–Ig3)_339−719_ under low-salt conditions. Ensembles initiated from NMR-consistent conformations provided the closest agreement with backbone and side-chain relaxation parameters, supporting their relevance as representations of experimentally populated states. Analysis of methyl dynamics across hydrophobic clusters revealed that fast side-chain motions remain largely unchanged between inactive and active-like ensembles, indicating that hydrophobic cores act as stabilising anchors while loop rearrangements drive functional transitions.

Together, these results support a dynamic model of MALT1 regulation in which coordinated loop rearrangements, modulated by ionic environment, govern access to catalytically competent states. Rather than existing as discrete static structures, MALT1 populates a conformational ensemble whose distribution is highly sensitive to electrostatic conditions. This dynamic framework provides a mechanistic basis for understanding how environmental factors, including physiological electrolytes, tune MALT1 activity and suggests new avenues for targeting its regulation through modulation of conformational dynamics.

## Methods

### Expression of isotope-labelled MALT1(PCASP-Ig3)_339–719_ and preparation of NMR samples

Expression, purification, and isotope labelling of MALT1(PCASP-Ig3)_339–719_ were performed as described previously^42^. Briefly, the construct (residues 339–719, C-terminal His6 tag) was expressed in *E. coli* using selective methyl labelling schemes and purified to monomeric homogeneity by affinity, ion-exchange, and size-exclusion chromatography. Final NMR samples were prepared in 10 mM Tris (pH 7.6), 50 mM NaCl, 2 mM TCEP, 0.002% NaN_3_, 10% D_2_O) and concentrated to 0.3–0.5 mM. Additional experimental details are provided in Supplementary S3.1.

### NMR relaxation experiments and data processing

All NMR experiments were performed at 298 K on Bruker 800 and 900 MHz spectrometers equipped with TCI cryogenic probes. Backbone and methyl relaxation data were acquired using established pulse sequences optimized for high-molecular-weight proteins.

Spectra were processed using NMRPipe and mddnmr and analysed using CcpNmr Analysis and Dynamics Center. Molecular graphics were prepared using UCSF Chimera. Detailed acquisition and processing parameters are provided in Supplementary S3.2.

#### Backbone^1^-^15^ N CSA/dipolar cross-correlated relaxation

Backbone^1^-^15^ N CSA/dipolar cross-correlated relaxation rates (ηₓ_γ_) were measured at 900 MHz using a modified version^46^ of the previously reported pulse sequences^47–49^. Experiments used eight relaxation delays ranging from 0 to 44 ms. Spectra were acquired with constant-time ^15^N evolution, and relaxation rates were obtained by mono-exponential fitting of peak intensities. Carrier frequencies were set to the water resonance for ^1^H and 118 ppm for ^15^N. Additional acquisition and processing parameters are provided in the Supplementary S3.2.1.

#### Methyl ^13^C-^1^H_3_ relaxation experiments

Methyl ^13^C–^1^H_3_ longitudinal (R_1_) and cross-correlated (Γ_2_) relaxation rates were measured at 800 MHz using interleaved pseudo-3D experiments as described previously^50,51^. R_1_ rates were extracted from mono-exponential fits of peak intensities recorded over relaxation delays ranging from 10 ms to 2 s.

Cross-correlated relaxation rates (Γ_2_) were measured using a constant-time experiment with multiple evolution delays. All experimental parameters followed established protocols^51,52^. Data were processed in TopSpin and analysed using Mathematica. Additional details are provided in Supplementary S3.2.2.

### Molecular dynamic simulation

Molecular dynamics (MD) simulations were used to investigate the conformational dynamics of monomeric MALT1(PCASP–Ig3)_339−719_ and the influence of ionic strength on loop-mediated transitions between inactive (PCASP-I) and active-like (PCASP-A) states. Simulations were initiated from both experimentally determined crystal structures and AF-derived models. Starting conformations encompassed combinations of PCASP-I and PCASP-A states with inward- or outward-facing orientations of residue W580, as defined by crystallographic references and AF predictions. Missing regions in crystal structures were modelled prior to simulation (Figure S9b). All starting conformations (Figure S9, S10) force fields, durations, and ionic conditions are summarized in Table 1 and Supplementary Methods S3.3.1-S3.3.5.

Trajectory grouping (Table 1): Trajectories 1–4: Low-salt conditions, CHARMM36/CUFIX, initiated from AF2/AF3 starting structures. Trajectories 5–6: Kosmotropic conditions, CHARMM36/CUFIX, initiated from AF3 and AF2 inactive and active-like structures. Trajectories 7–9: High-salt conditions, CHARMM36/CUFIX, initiated from inward- or outward-facing W580 structures. Trajectories 10–11: Low-salt conditions, CHARMM36/CUFIX, initiated from experimentally determined X-ray inactive structures. Trajectory 12: Low-salt conditions, polarizable AMOEBA force field, initiated from AF3inactive structure.

All production simulations used the CHARMM36 all-atom force field with CUFIX corrections in GROMACS (v2023.1). The protein was simulated at 298 K and pH 7.6. The NMR experiments were performed in buffer containing 50 mM NaCl. In MD simulations, the low ionic strength was set to ~ 60 mM by explicitly adding ions, which accounts for both the nominal salt concentration and additional ionic strength contributions from buffer components and residual ions present in experimental conditions. In total three ionic environments were examined: 60 mM NaCl (low ionic strength), intermediate kosmotropic conditions (166.7 mM trisodium citrate, as used in in vitro activity assays) and high ionic strength (500 mM NaCl).

Because the simulations were designed to reproduce the in vitro experimental conditions used in NMR and activity assays^43,44^, NaCl was used rather than KCl. The results therefore primarily reflect the effect of ionic strength and electrostatic shielding under these conditions. We note, however, that ion-specific effects cannot be excluded and are not explicitly addressed in the present study.

These conditions were chosen to systematically modulate electrostatic shielding and assess its impact on conformational equilibria.

Each system was energy-minimized, equilibrated, and simulated for microsecond timescales under periodic boundary conditions. Multiple independent trajectories were generated to assess convergence and reproducibility. Structural stability and conformational sampling were monitored using root-mean-square deviation (RMSD), principal component analysis (PCA), and clustering.

To assess whether explicit electronic polarization is required to reproduce experimentally observed dynamics at low ionic strength, a subset of the simulation was additionally performed using the polarizable AMOEBA force field (trajectory 12). Comparison of back-calculated NMR relaxation parameters with experimental data showed no improvement relative to CHARMM36/CUFIX. Accordingly, the non-polarizable CHARMM36/CUFIX force field was used for all analysis presented (Supplementary S3.3.3).

Detailed simulation parameters, equilibration protocols, force-field benchmarking, and ion parameterization are provided in Supplementary S3.3.2-S3.3.4.

### Identifying stable conformational ensembles from molecular dynamics simulations

**Fig. 8:**
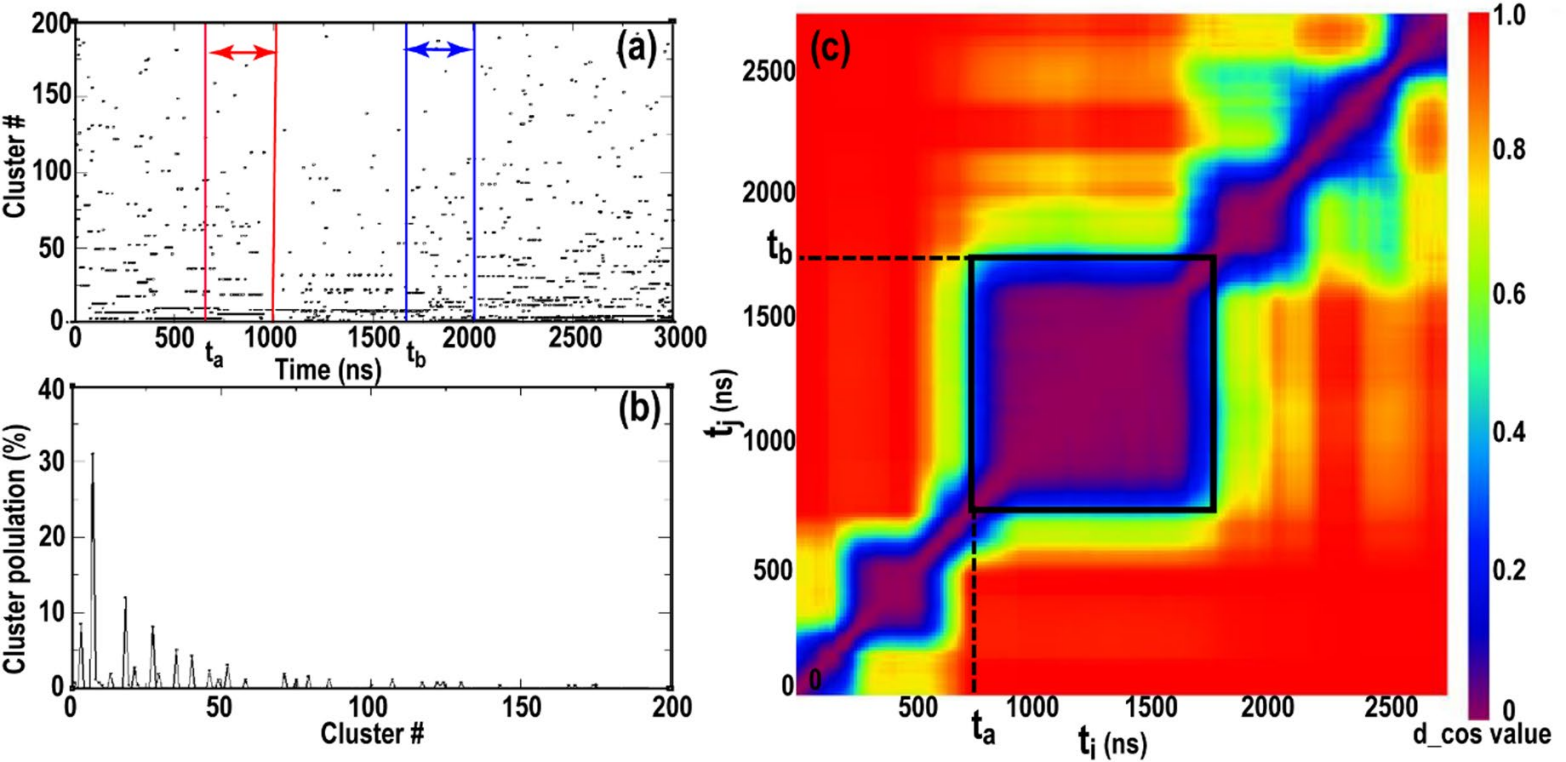
Identification of a stable conformational ensemble in MD trajectory. (**a**) Distribution of frames from MD trajectory 1, sampled every 1 ns, across structural clusters. As an example, time windows of duration 10τc, initiated at t_i_ = tₐ and tⱼ = t_b_, are indicated by red and blue bars, respectively. (**b**) Cluster population distributions for the time window [tₐ, tₐ + 10τ_c_], represented as a population vector $\:\overrightarrow{\mathbf{a}}\left({\mathbf{t}}_{\mathbf{i}}\right)$, whose components correspond to the fractional occupancy of individual clusters. (**c**) Heat map of pairwise cosine distances between cluster population vectors for all time windows derived from MD trajectory 1. The temporally stable region selected for subsequent analysis is outlined by a black square, spanning from tₐ = 750 ns to t_b_ = 1785 ns, corresponding to a total duration of t_b_ − tₐ + 10τ_c_ = 1292 ns.

Stable conformational ensembles persisting longer than 10 rotational correlation times (τ_c_) were identified using a time-resolved clustering and population-based analysis. The procedure is illustrated in Fig. 8 and was applied independently to each MD trajectory.

Each trajectory was first down sampled at 1 ns intervals, yielding a set of discrete structural frames (for example, a 3000 ns trajectory yields 3000 frames; Fig. 8a). All frames were subjected to structural clustering using the GROMOS algorithm with a backbone RMSD cutoff of 0.1 nm, such that the pairwise RMSD between any two frames within a cluster was ≤ 0.1 nm. This step assigned every frame to the structural cluster.

To assess temporal stability, the trajectory was then partitioned into overlapping time windows of duration 10τc, advanced by 15 ns increments between successive windows (t_i_₊_1_ = t_i_ + 15 ns). For each time window starting at time t_i_, the population of each cluster was recalculated using the preassigned cluster identities of the frames within that window (Fig. 8b). This yielded a cluster population vector $\:\overrightarrow{\mathbf{a}}\left({\mathbf{t}}_{\mathbf{i}}\right)$ = [x_1_, x_2_, …, x_n_], where x_k_ denotes the percentage of frames in the window assigned to the k-th cluster, including clusters with zero population.

Similarity between cluster population vectors corresponding to different time windows was quantified using the cosine distance metric^50^:

1 $$\:\begin{array}{cccc}&\:\mathbf{d}(\overrightarrow{\mathbf{a}},\overrightarrow{\mathbf{b}})=1-\frac{\overrightarrow{\mathbf{a}}\cdot\:\overrightarrow{\mathbf{b}}}{\mid\:\mathbf{a}\mid \mid\:\mathbf{b}\mid\:}&\:&\:\end{array}$$

where $\:\overrightarrow{\mathbf{a}}$ and $\:\overrightarrow{\mathbf{b}}$ represent cluster population vectors for windows starting at times tₐ and t_b_, respectively. A cosine distance of 0 indicates identical cluster population distributions, whereas a value of 1 indicates complete dissimilarity. Pairwise cosine distances between all time windows were visualized as a heat map (Fig. 8c).

A stable conformational ensemble was defined by the presence of a contiguous square region along the diagonal of the cosine-distance matrix in which all pairwise distances were below a threshold of 0.1^51^. Such a region corresponds to a time interval spanning from tₐ to t_b_, within which the cluster population distributions remain invariant. As the analysis is performed using windows of duration 10τ_c_, the final trajectory segment representing the stable ensemble was defined as the interval from tₐ to t_b_, + 10τ_c_.

### Trajectory analysis and back-calculation of NMR observables

MD trajectories were analysed to characterise global conformational stability, dominant collective motions, and agreement with experimental NMR relaxation data. Structural convergence and conformational sampling were assessed using RMSD, PCA, RMSF and clustering of backbone conformations. For reference throughout this study, trajectories are grouped as follows: 1–4 correspond to low-salt conditions, 5–6 to kosmotropic citrate, 7–9 to high-salt, 10–11 to X-ray starting structures, and 12 to simulations performed with the polarizable AMOEBA force field.

RMSD analysis were performed on backbone heavy atoms after alignment to reference structures, excluding terminal regions and highly flexible loops. PCA was applied to backbone coordinates to identify dominant collective motions and visualise conformational distributions across trajectories initiated from different starting structures and ionic conditions (Supplementary S3.4.1).

For quantitative validation against experimental data, backbone ^15^N and side-chain methyl and ^13^C relaxation parameters were back-calculated from equilibrated trajectory segments and compared with experimentally measured values. Back-calculated parameters included longitudinal (R_1_) and transverse relaxation rates (R_2_), ^1^-^15^ N heteronuclear NOEs, and CSA/dipole cross-correlated relaxation rates (η_xy_) (Supplementary S3.4.2, Figure S8).

Agreement between simulated and experimental data was quantified using multiple complementary scoring metrics, including cosine similarity, mean absolute error (MAE), and root-mean-square error (RMSE). Trajectories were ranked according to overall agreement, with experimental relaxation data, allowing identification of MD ensembles that best represent the dominant solution-state dynamics of MALT1(PCASP–Ig3)_339−719_. Detailed analysis procedures, equations, and statistical treatments are described in the Supplementary S3.5 and data presented in Table S2.

## Supplementary Information

Below is the link to the electronic supplementary material.

Supplementary Material 1

## Data Availability

The assignment is found in BiologicalMagneticResonanceDataBank (http://bmrb.pdbj.org/) with the BMRB accession code 52265. The numerical sources for the graphs and plots are found in the supplementary data. The 1-st trajectory under low-salt conditions (60 mM NaCl; 2500-3000 ns time interval) was clustered into 20 states using a RMSD cutoff of 0.105 nm. The cluster populations were: 1–34.4%, 2–17.7%, 3–13.5%, 4–8.9%, 5–4.4%, 6–3.5%, 7–3.3%, 8–2.7%, 9–1.6%, 10–1.3%, 11–1.0%, 12–1.0%, 13–0.9%, 14–0.7%, 15–0.6%, 16–0.5%, 17–0.5%, 18–0.5%, 19–0.4%, 20–0.3%, and other–2.3%. The most representative 20 structures were selected to construct a conformational ensemble, which was deposited in the Protein Data Bank (PDB ID: 9AAC). The 9-th trajectory under high-salt conditions (500 mM NaCl; 3500-4000 ns time interval) was clustered into 20 states using a RMSD cutoff of 0.105 nm. The cluster populations were: 1–43.8%, 2–24.2%, 3–6.7%, 4–6.2%, 5–4.5%, 6–2.6%, 7–2.3%, 8–1.9%, 9–0.9%, 10–0.9%, 11–0.9%, 12–0.8%, 13–0.8%, 14–0.7%, 15–0.5%, 16–0.4%, 17–0.4%, 18–0.3%, 19–0.2%, 20–0.2%, and other–0.9%. The most representative 20 structures were selected to construct a conformational ensemble, which was deposited in the Protein Data Bank (PDB ID: 9AAD).
