# Supplementary material for: Loop dynamics govern MALT1 activation revealed by integrative AlphaFold, MD, and NMR analysis
Source: Sci Rep. 2026 May 20;16:15709. doi: 10.1038/s41598-026-53505-4 (PMC13190690; doi:10.1038/s41598-026-53505-4)
Supplement: Supplementary file 1 — Supplementary Material 1 [file 41598_2026_53505_MOESM1_ESM.docx]

**Supporting Information**

**Loop dynamics govern MALT1 activation revealed by integrative AlphaFold, MD, and NMR analysis**

Dmitry Lesovoy^1#^, Tatiana Agback^2,3#^, Konstantin Roshchin^1^, Tatyana Sandalova^4,5^, Adnane Achour^4.5^, Xiao Han^4,5^, Alexander Lomzov^6^, Vladislav Orekhov^2,7*^, Peter Agback^2*^.

^1^Shemyakin-Ovchinnikov Institute of Bioorganic Chemistry RAS, 117997 Moscow, Russia.

^2^Department of Molecular Sciences, Swedish University of Agricultural Sciences, PO Box 7015, SE-750 07 Uppsala, Sweden.

^3^Department of Chemistry and Molecular Biology, University of Gothenburg, Box 465, SE-40530

^4^Science for Life Laboratory, Department of Medicine, Solna, Karolinska Institute SE-17165 Solna

^5^Division of Infectious Diseases, Karolinska University Hospital, SE‑171 76 Stockholm, Sweden.

^6^Laboratory of Structural Biology, Institute of Chemical Biology and Fundamental Medicine SB RAS, 630090 Novosibirsk, Russia.

^7^Swedish NMR Centre, Science for Life Laboratory, SciLifeLab, University of Gothenburg, Box 465, SE-40530 Gothenburg, Sweden.

* Corresponding authors: [peter.agback@slu.se](mailto:peter.agback@slu.se); vladislav.orekhov@nmr.gu.se

^#^ These authors contributed equally to this work.

**Table of Contents**

1. **S1.1 Figures**
   1. **Figure S1** Structural superpositions along the first principal component under different ionic conditions.
   2. **Figure S2** Conformational dynamics of MALT1(PCASP–Ig3)_339-719_ under different ionic conditions.
   3. **Figure S3** Ionic-strength dependence of backbone amide dynamics in MALT1(PCASP–Ig3)_339–719._
   4. **Figure S4** Dependence of backbone amide dynamics of MALT1(PCASP–Ig3)_339-719_ on starting conformations at low ionic strength.
   5. **Figure** **S5** Methyl-group relaxation dynamics of MALT1(PCASP–Ig3)_339–719_ measured at 800 MHz.
   6. **Figure S6.** Methyl relaxation dynamics across hydrophobic clusters in MALT1(PCASP–Ig3)_339–719_.
   7. **Figure S7** Force-field effects on backbone amide dynamics of MALT1(PCASP–Ig3)_339–719_
   8. **Figure S8** Examples of backbone NH autocorrelation decay in MALT1(PCASP–Ig3)_339–719_.
   9. **Figure S9** X ray structures of MALT1
   10. **Figure S10** AF2 and AF3 structures of MALT1 used as starting structures for MD calculations
2. **S1.2 Tables**
   1. **Table S1.** The first three eigenvalues for the first three principal components (PCs) for trajectories.
   2. **Table S2.** Penalty functions
3. **S2.0 Result**
   1. **S2.1** Analysis of trajectory 4
   2. **S2.2** Analysis of trajectory 3
   3. **S2.3** Analysis of trajectory 1
   4. **S2.4** Analysis of trajectory 2
   5. **S2.5** Analysis of trajectories 8, 9
   6. **S2.6** Methyl Group Dynamics as Probes of Conformational Stability: Agreement Between MD and NMR
   7. **S2.7** Relaxation Analysis Across Hydrophobic Clusters
      1. Cl2: A Critical Cluster at the Ig3–PCASP Interface
   8. **S2.8** Backbone Dynamics of MALT1(PCASP–Ig3)_339-719_ Conformation
4. **S3 Methods**

## **S3.1** Expression of isotope-labelled MALT1(PCASP-Ig3)_339–719_ and preparation of NMR samples

## **S3.2** NMR relaxation experiments and data processing

## **S3.2.1** Determination of ^1^H-^15^N CSA/dipole cross correlation (η_xy_) relaxation

## **S3.2.2** Methyl ¹³C–¹H₃ relaxation experiments

## **S3.3** Molecular Dynamic simulation

## **S3.3.1** Starting structures for MD-simulations

## **S3.3.2** The Protocol used in MD Simulations

## **S3.3.3** MD Simulations Using the Non-Polarizable CHARMM36 Force Field

## **S3.3.4** Molecular Dynamics Simulations Using the Polarizable AMOEBA Force Field

- 1. **S3.4** MD Trajectory Analysis: Alignment, RMSD Calculation, and Back-Calculation of NMR Relaxation Parameters
     1. S3.4.1 Alignment, RMSD, PCA
     2. S3.4.2 Individual MD trajectory analysis with back-calculation of theoretical ^15^N and ^13^C relaxation parameters
  2. **S3.5** Penalty functions for MD trajectory validation

## **References**

**S1.1 Figures**

**
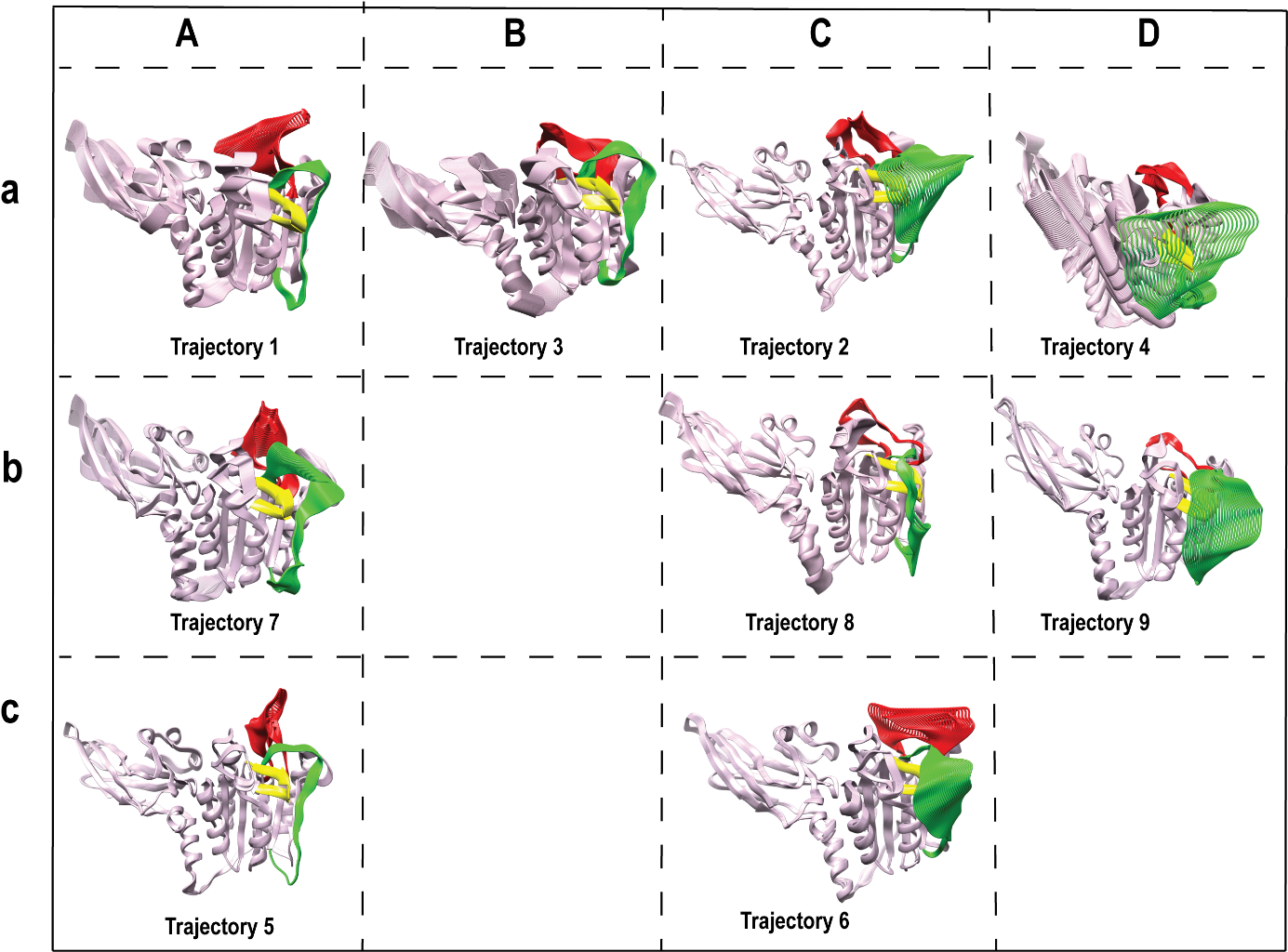
**

**Figure S1 Structural superpositions along the first principal component under different ionic conditions.** Superpositions of 30 structures sampled from opposite extremes of the conformational space defined by the first principal component (PC1) for all ensembles. Columns A and B show structures in the inactive conformation, whereas columns C and D show structures in the active conformation. Columns A and C correspond to the inward-facing orientation of W580, while columns B and D correspond to the outward-facing W580 orientation. Rows (a–c) represent, respectively, 60 mM NaCl (55 Na^+^ 41 Cl^-^); 500 mM NaCl (415 Na⁺, 401 Cl⁻); 166.7 mM sodium citrate (416 Na⁺, 134 C₆H₅O₇³⁻). Colours coding highlights the β3 hairpin (residues 416–425), loop 2 (464–485), loop 3 (491–509).


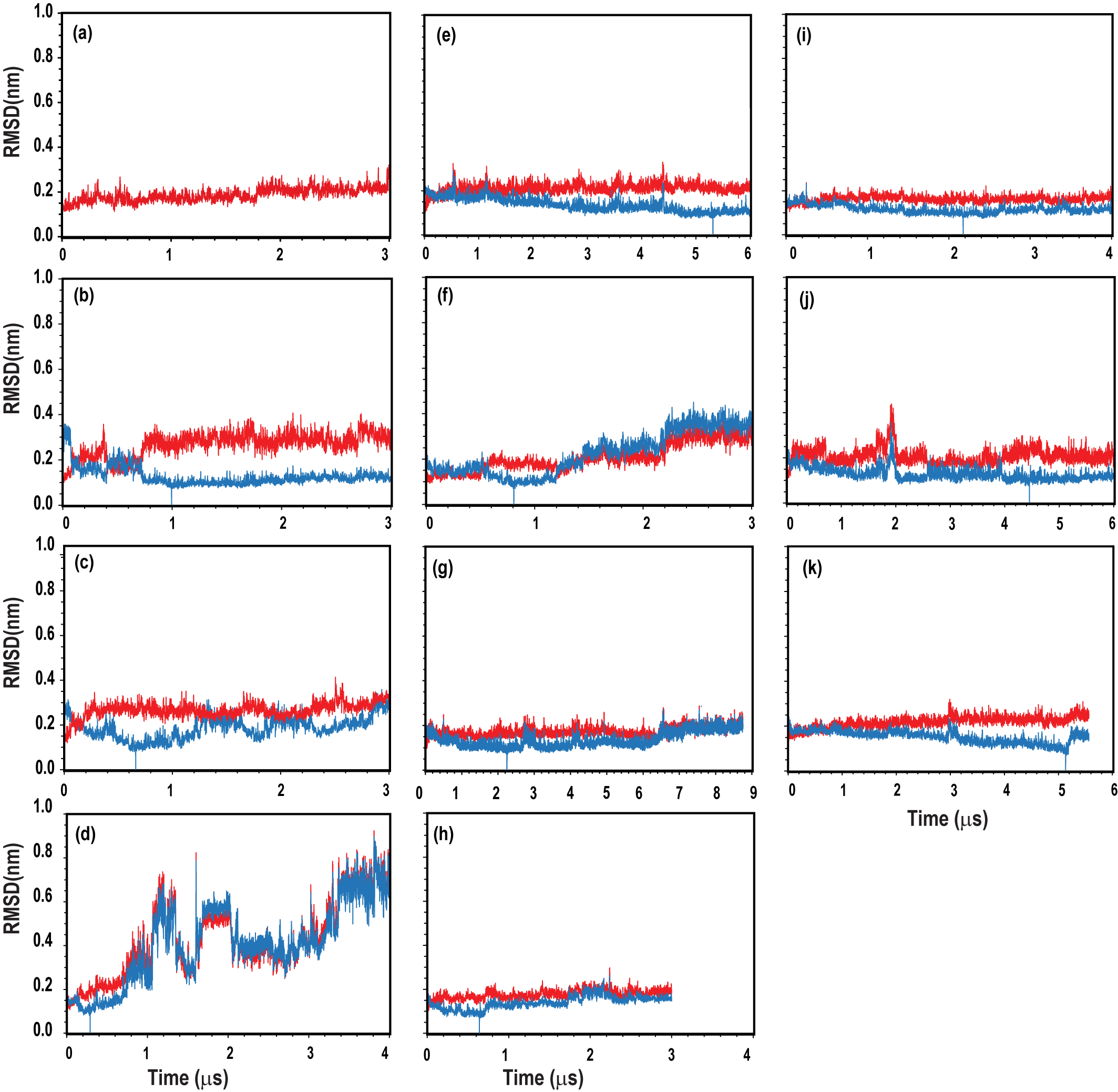


**Figure S2 Conformational dynamics of MALT1(PCASP–Ig3)_339-719_ under different ionic conditions.** Root-mean-square deviation (RMSD) of individual molecular dynamics trajectories relative to their respective starting structures (red) and representative structure of the most populated cluster (blue). Panels (a–f) correspond to simulations at low ionic strength (60 mM NaCl; 55 Na^+^ 41 Cl^-^) and show; trajectories **10**, **1**, **12**, **4**, **2** and **3**, respectively. Panels (g–i) correspond to high ionic strength conditions (500 mM NaCl; 415 Na⁺, 401 Cl⁻) and show trajectories **7**, **8** and **9**, respectively. Panels (j–k) correspond to kosmotropic conditions using sodium citrate (166.7 mM, 416 Na⁺, 134 C₆H₅O₇³⁻) and show trajectories **5** and **6**, respectively.


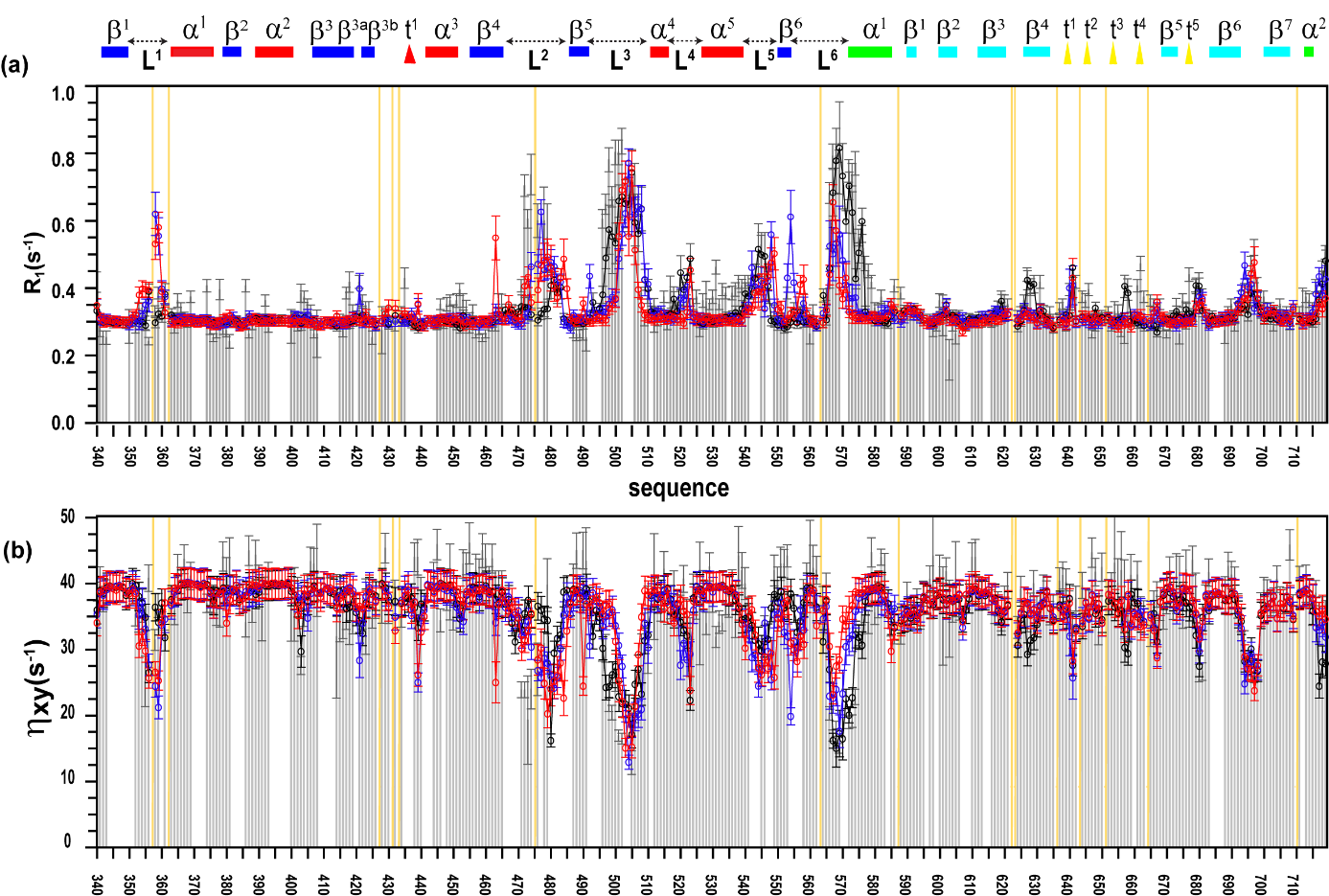


**Figure S3 Ionic-strength dependence of backbone amide dynamics in MALT1(PCASP–Ig3)_339–719._** Backbone ^1^H-^15^N amide relaxation parameters of MALT1(PCASP–Ig3)_339-719_ measured at 900 MHz. Longitudinal relaxation rates R_1_ (s⁻¹) and CSA/dipole cross-correlation relaxation rates η_xy_ (s⁻¹) are shown for ensembles **8**, **9** and **1**. Experimentally measured R_1_ and η_xy_ values are shown as light-grey bars. Corresponding parameters predicted from molecular dynamics ensembles **8**, **9** and **1** are shown as solid blue, red and black lines, respectively. Ensembles **8** and **9** were generated from trajectories segments 2500–3000 ns and 3500–4000 ns, respectively, at high ionic strength (500 mM NaCl; 415 Na⁺, 401 Cl⁻) using the using the CHARMM36/CUFIX force field. Ensemble **1** was generated from trajectory segment 2500–3000 ns at low ionic strength (60 mM NaCl; 55 Na⁺, 41 Cl⁻) using the same force field. Experimental error bars represent one standard deviation from curve fitting, and uncertainties in predicted parameters were estimated by bootstrap analysis.

**
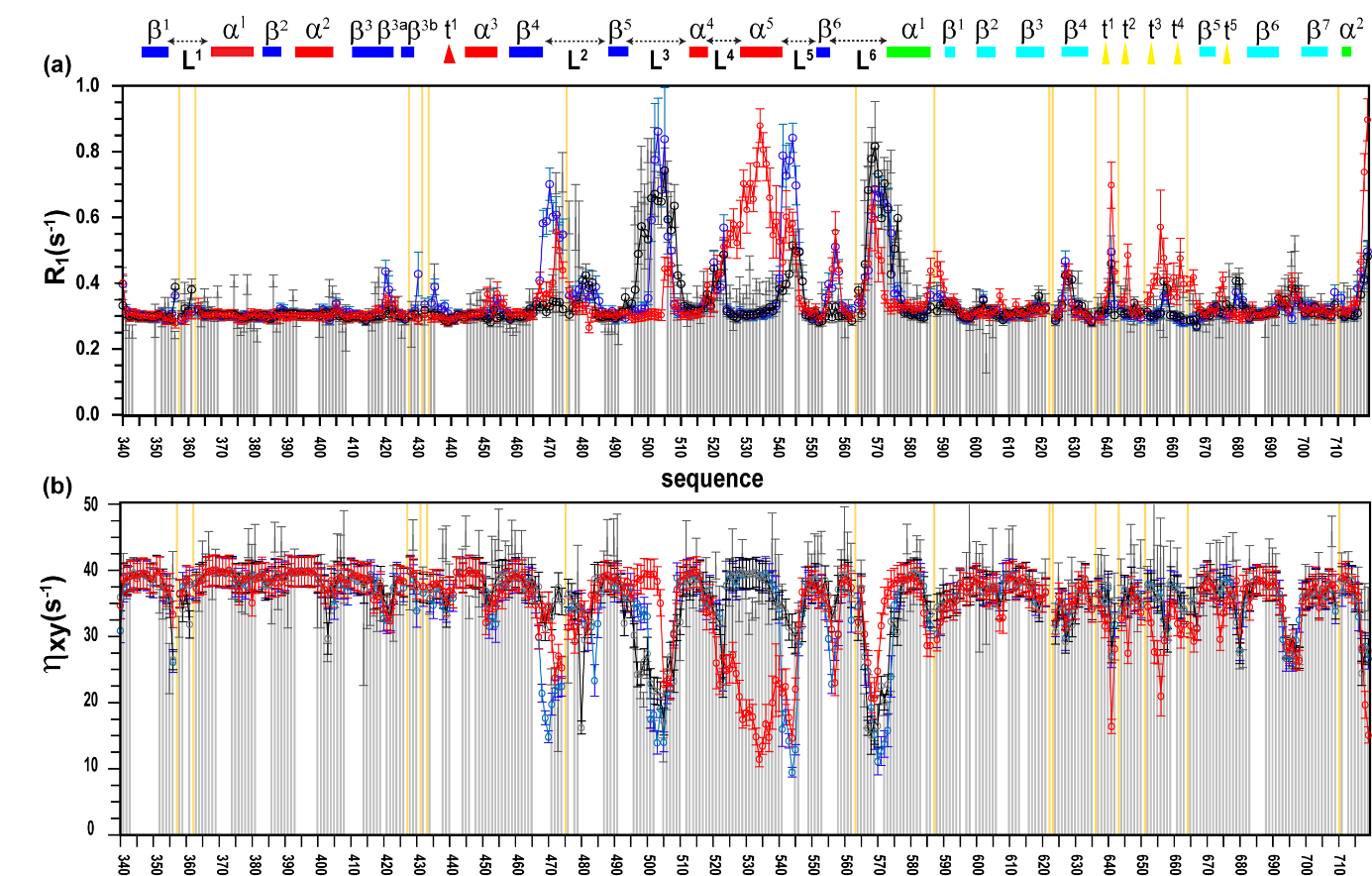
**

**Figure S4 Dependence of backbone amide dynamics of MALT1(PCASP–Ig3)_339-719_ on starting conformations at low ionic strength.** Backbone ^1^H-^15^N amide relaxation parameters of MALT1(PCASP–Ig3)_339-719_ measured at 900 MHz. Longitudinal relaxation rates R_1_ (s⁻¹) (panel a) and CSA/dipole cross-correlation relaxation rates η_xy_ (s⁻¹) (panel b) are shown for ensembles **10**, **1,** and **11** which were generated from molecular dynamics trajectories initiated from distinct starting structures V, I, and VI, respectively (**Table 1**). Experimentally measured R_1_ and η_xy_ values are shown as light-grey bars. Corresponding parameters predicted from molecular dynamics ensembles **10**, **1,** and **11** are shown as solid blue, black, and red lines, respectively. All ensembles were generated from trajectory segments 2500–3000 ns at low ionic strength (60 mM NaCl; 55 Na⁺, 41 Cl⁻) using the CHARMM36/CUFIX force field. Experimental error bars represent one standard deviation from curve fitting, and uncertainties in predicted parameters were estimated by bootstrap analysis.


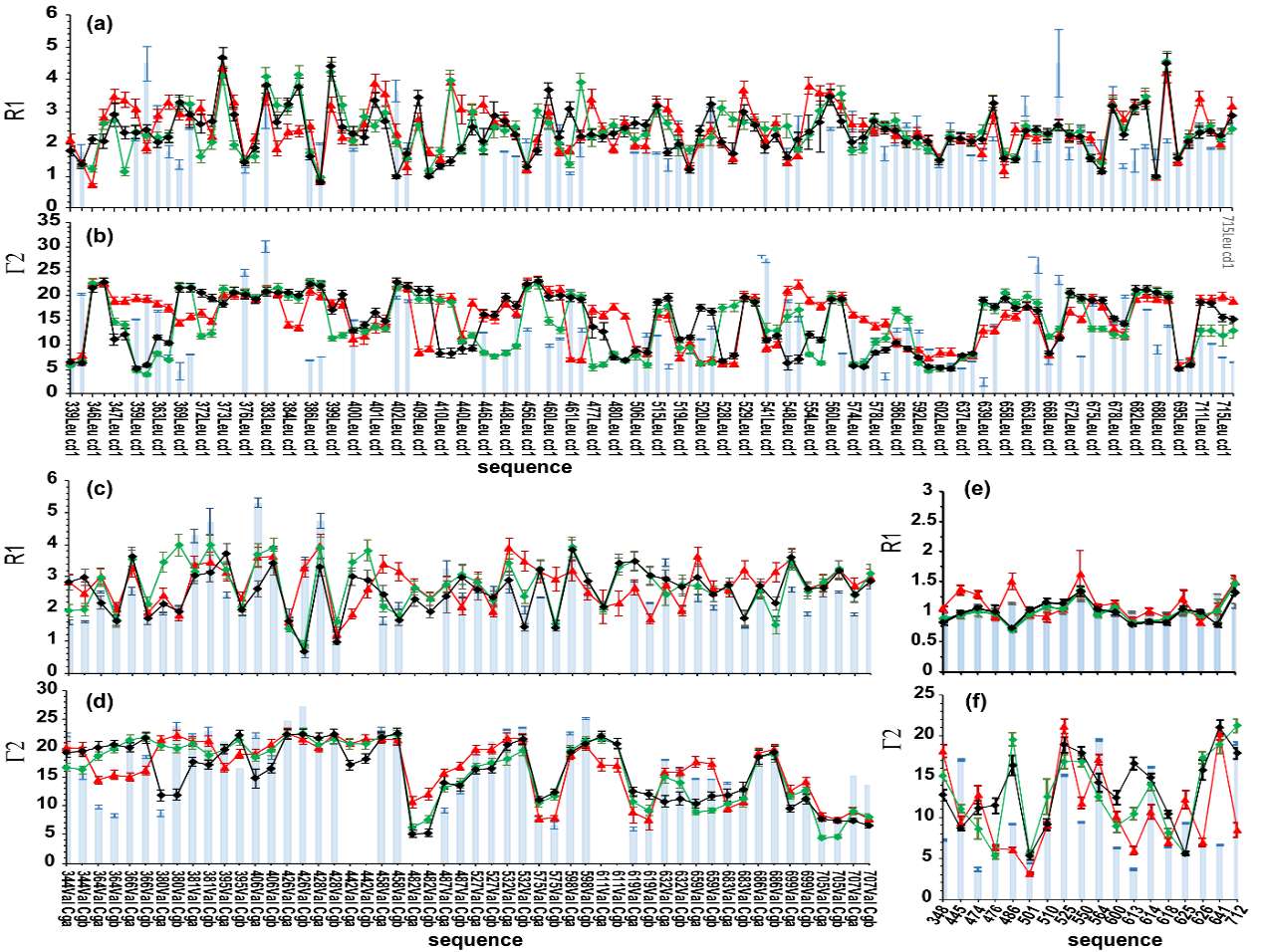


**Figure S5 Methyl-group relaxation dynamics of MALT1(PCASP–Ig3)_339–719_ measured at 800 MHz.** Experimental and back-calculated methyl relaxation parameters of isoleucine (Ile), leucine (Leu), and valine (Val) residues are shown. Longitudinal relaxation rates R_1_ (s^-1^) and transverse relaxation rates Γ_2_ (s^-1^) are presented for Leu (panels a, b), Val (panels c, d), and Ile (panels e, f). Experimental data are shown in light blue, while values back-calculated from molecular dynamics trajectories **1**, **8**, and **9** are shown in red, green, and black, respectively.


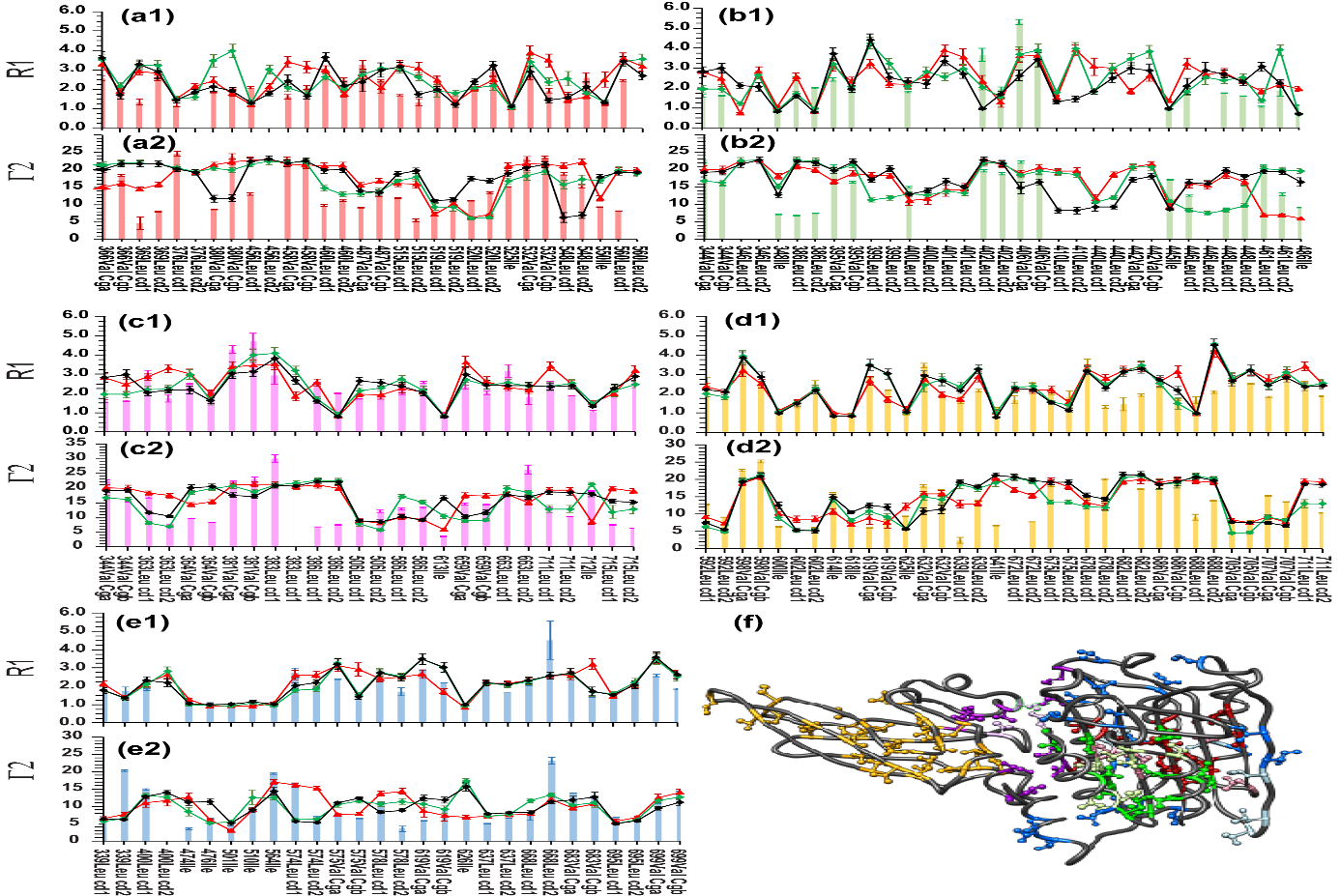


**Figure S6**. **Methyl relaxation dynamics across hydrophobic clusters in MALT1(PCASP–Ig3)_339–719_.** (a1–e1) Experimental R_1_ (s^-1^) relaxation rates measured at 800 MHz, and 25 °C grouped by hydrophobic clusters: Cl3 (a1), Cl4 (b1), Cl2 (c1), Cl1 (d1), and surface-exposed methyl groups (e1). (a2–e2) Corresponding experimental Γ_2_ (s^-1^) relaxation rates. Experimental values are shown as colour-coded brackets matching the cluster colours in panel (f). Back-calculated relaxation parameters from molecular dynamics trajectories are shown as solid lines: trajectory **1** (red), trajectory **8** (green), and trajectory **9** (black). (f) Spatial distribution of methyl clusters mapped onto the MALT1 structure. Cl1 (yellow) is located within the Ig3 domain; Cl2 (violet) at the Ig3–PCASP interface; Cl3 (green) and Cl4 (red) lie on opposite faces of the PCASP β-sheet. Surface-exposed methyl groups not assigned to clusters are shown in blue. Assigned methyl groups are indicated with darker shades, whereas unassigned methyl groups are shown in lighter shades. Experimental error bars represent one standard deviation from curve fitting; uncertainties in calculated parameters were estimated by bootstrap analysis.


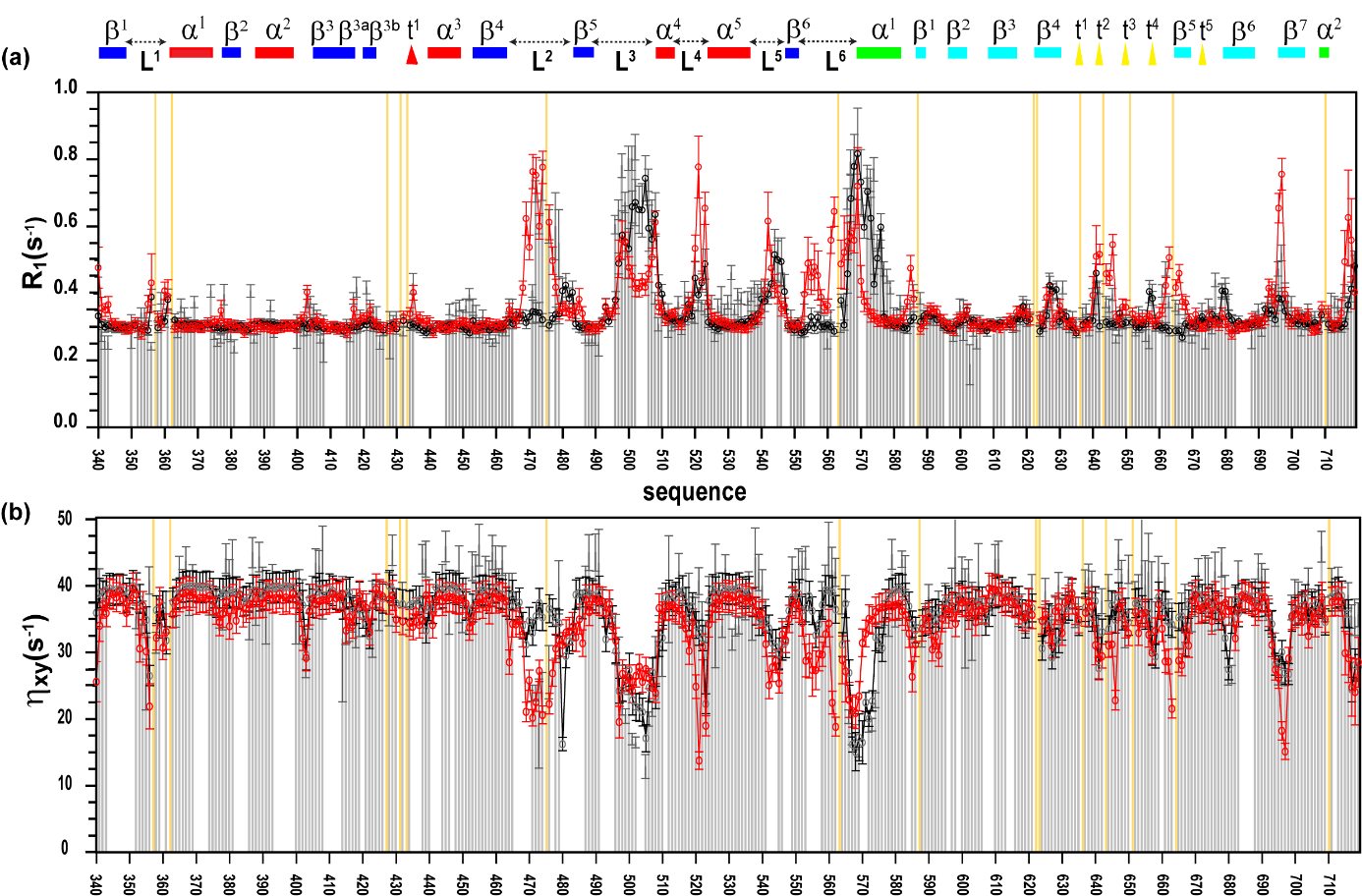


**Figure S7 Force-field effects on backbone amide dynamics of MALT1(PCASP–Ig3)_339–719_**

Backbone ^1^H-^15^N amide relaxation parameters measured at 900 MHz are shown. Panels (a) and (b) display longitudinal relaxation rates R_1_(s^−1^) and CSA/dipole cross-correlation rates η_xy_ (s^−1^), respectively. Experimental values are shown as light-grey bars. Predicted values from molecular dynamics ensembles derived from trajectory **1** (CHARMM36 force field; black lines) and trajectory **12** (AMOEBA force field; red lines) are shown as solid lines. Both ensembles were generated from trajectories segments 2500–3000 ns. Error bars represent one standard deviation for experimental data and bootstrap-derived uncertainties for predicted parameters. Proline residues are indicated by yellow bars.


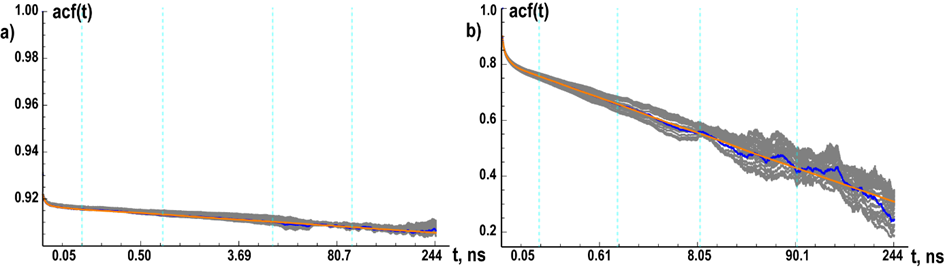


**Figure S8 Examples of backbone NH autocorrelation decay in MALT1(PCASP–Ig3)_339–719_.** Representative backbone NH autocorrelation functions (acf) are shown for residues 365D (a) and 571A (b), illustrating the decay over time (ns). The fitted approximation, residue-averaged values, and deviations from MD simulations are shown in orange, blue, and grey, respectively. Blue dashed lines indicate the acf(t) values at 0.1 ns, 1 ns, 10 ns, and 100 ns for the relatively stable NH vector of 365D (a) and the more flexible NH vector of 571A (b).

**
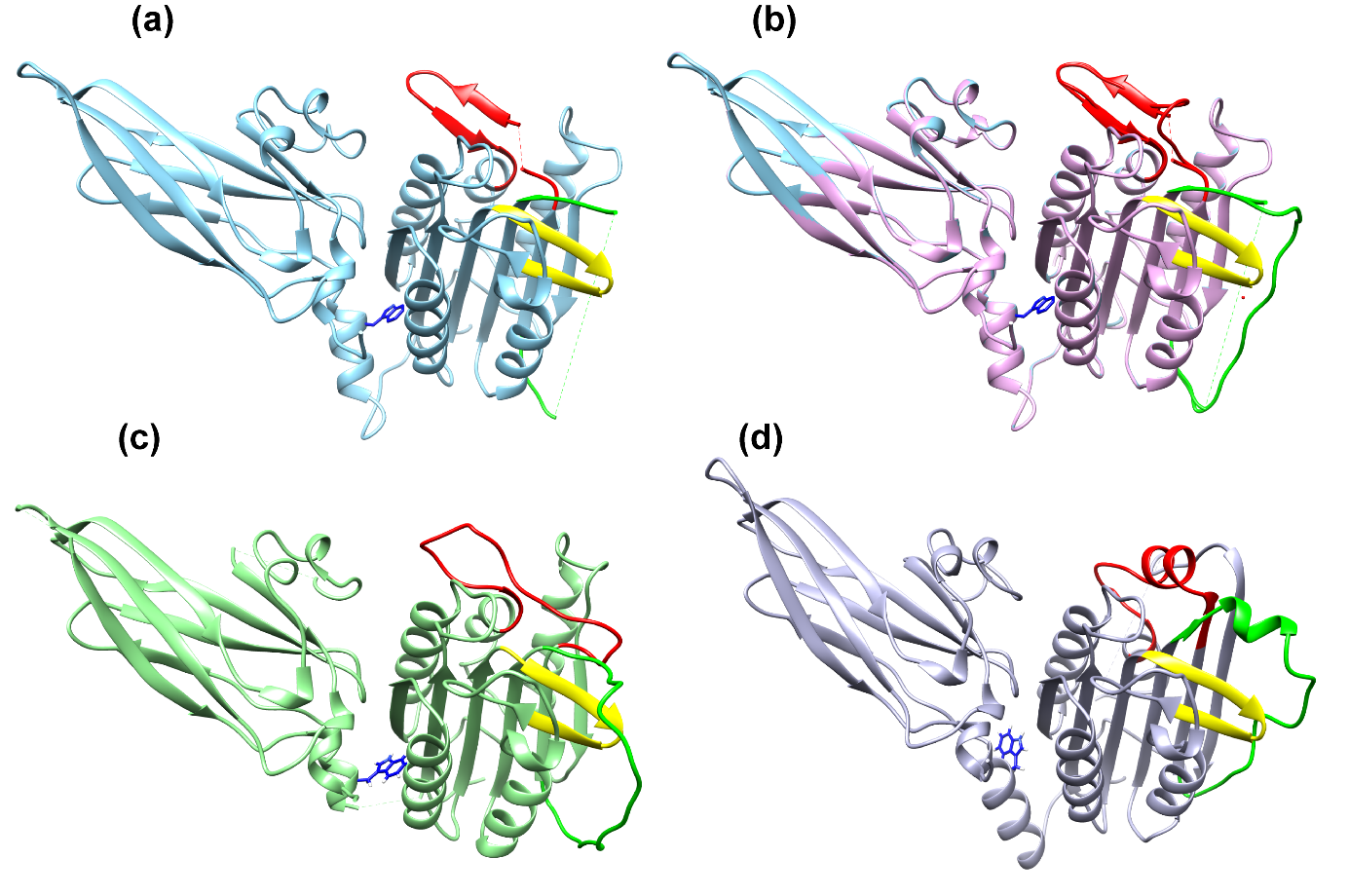
**

**Figure S9 X ray structures of MALT1**

MALT1 structures presented as ribbons for (a) PDB 3V55; (b) Superpositions of PDB 3V55 with corresponding structure where loop 2 (green) has been added with PDBFixer ^20^ after AF simulation; (c) PDB 3uo8 and (d) PDB 9MKD. Structure (c) and (d) one crystallized as dimer but shown only one domain. Colours coding highlights the β3 hairpin (residues 416–425), loop 2 (464–485), loop 3 (491–509), and the orientation of W580 (inward or outward, in blue).


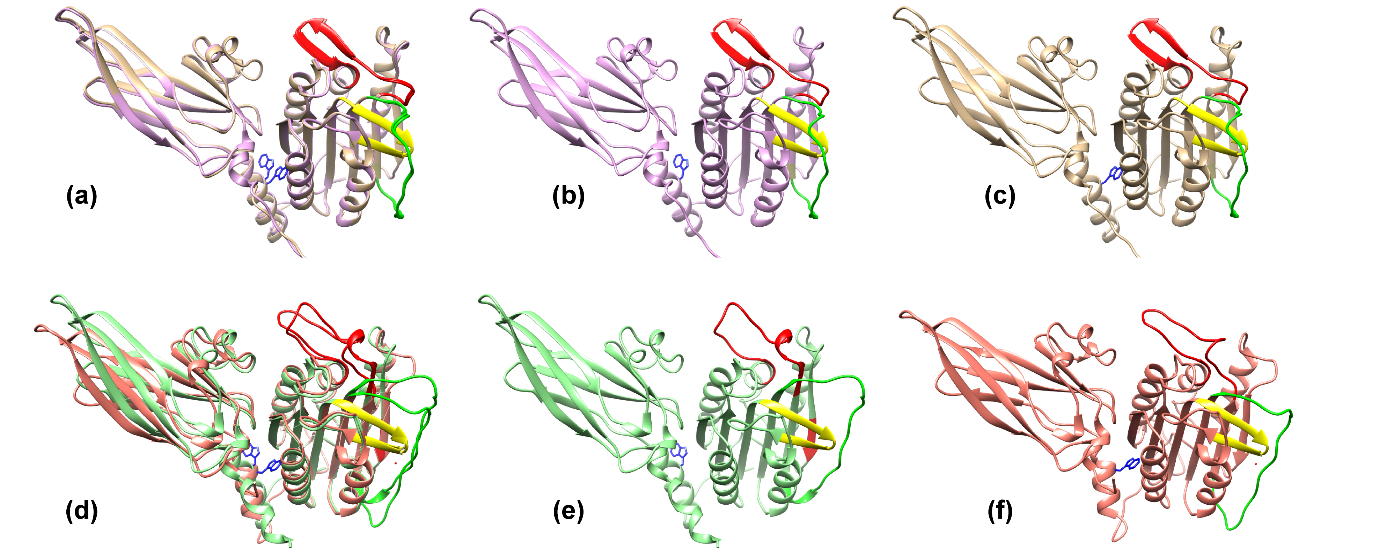


**Figure S10 AF2 and AF3 structures of MALT1 used as starting structures for MD calculations**

(a) Superposition of the MALT1 structures with active conformation of PCASP domains: (b) IV: AF2 active, W580 outward; (c) II: AF2 active, W580 inward. (d) Superposition of the MALT1 structures with inactive conformation of PCASP domains: (e) III: AF3 inactive, W580 outward, (f) I: AF3 inactive, W580 inward). Colours coding highlights the β3 hairpin (residues 416–425), loop 2 (464–485), loop 3 (491–509), and the orientation of W580 (inward or outward, in blue).

**S1.2 Tables**

**Table S1.** Eigenvalues of the first three principal components for all MD trajectories.

| № MD trajectory | Backbone heavy-atoms of stable regions 1^a^ | | | Backbone heavy-atoms of stable regions 2^b^ | | |
| --- | --- | --- | --- | --- | --- | --- |
|  | PC1^c^ | PC2 | PC3 | PC1 | PC2 | PC3 |
| 1 | 19.3 | 3.2 | 2.3 | 7.5 | 1.6 | 1.1 |
| 2 | 20.7 | 3.2 | 2.6 | 3.6 | 1.9 | 1.1 |
| 3 | 32,8 | 4,8 | 3,5 | 21,4 | 2,4 | 1,7 |
| 4 | 103.1 | 59.7 | 28.5 | 65.1 | 51.7 | 20.8 |
| 6 | 20.9 | 7.7 | 5.2 | 4.1 | 2.0 | 1.7 |
| 8 | 4.9 | 3.5 | 1.6 | 3.0 | 2.8 | 1.4 |
| 9 | 8.2 | 2.9 | 1.7 | 2,5 | 1,4 | 0,8 |
| 10 | 7.4 | 3.9 | 2.5 | 3.6 | 1.8 | 1.2 |
| 11 | 11.2 | 8.2 | 5.9 | 5.5 | 3.6 | 2.3 |
| 12 | 12.7 | 6.9 | 4.4 | 6.7 | 4.0 | 2.8 |

^a^ region used to perform PCA: 342-717

^b^ regions used to perform PCA: 342-467, 484-491, 509-562, 572-717

^c^ λ eigenvalue for each PC_i_ (nm^2^)

**Table S2.** Penalty function values for MD-predicted NMR relaxation parameters

| # tr^a^ /RP^b^ | R_1_ (900MHz) | R_2_ (900MHz) | (η_xy_) (900MHz) | R_1_ (800MHz) | NOE (800MHz) |
| --- | --- | --- | --- | --- | --- |
| 1^c^ | 0.1897 | 0.1195 | 0.1396 | 0.2368 | 0.2044 |
| 1^d^ | 0.2919 | 0.1659 | 0.1905 | 0.3416 | 0.1905 |
| 1^e^ | 0.0321 | 0.0118 | 0.0168 | 0.0400 | 0.0343 |
| 8^c^ | 0.2316 | 0.1361 | 0.1619 | 0.2599 | 0.1837 |
| 8^d^ | 0.3542 | 0.1735 | 0.2127 | 0.3853 | 0.2127 |
| 8^e^ | 0.0449 | 0.0136 | 0.0214 | 0.0499 | 0.0307 |
| 9^c^ | 0.2467 | 0.1390 | 0.1700 | 0.2788 | 0.1927 |
| 9^d^ | 0.3721 | 0.1871 | 0.2319 | 0.4040 | 0.2319 |
| 9^e^ | 0.0494 | 0.0166 | 0.0257 | 0.0565 | 0.0309 |
| 10^c^ | 0.2016 | 0.1315 | 0.1416 | 0.2435 | 0.1818 |
| 10^d^ | 0.3170 | 0.1756 | 0.1894 | 0.3583 | 0.1894 |
| 10^e^ | 0.0396 | 0.0131 | 0.0162 | 0.0468 | 0.0296 |
| 11^c^ | 0.2513 | 0.1600 | 0.1865 | 0.2822 | 0.1934 |
| 11^d^ | 0.3997 | 0.2191 | 0.2524 | 0.4243 | 0.2524 |
| 11^e^ | 0.0689 | 0.0227 | 0.0304 | 0.0740 | 0.0320 |
| 12^c^ | 0.2302 | 0.1676 | 0.1802 | 0.2564 | 0.2259 |
| 12^d^ | 0.3450 | 0.2196 | 0.2322 | 0.3651 | 0.2322 |
| 12^e^ | 0.0536 | 0.0186 | 0.0229 | 0.0576 | 0.0392 |

^a^ MD trajectory number (as in Table 1)

^b^ NMR Relaxation parameter and NMR spectrometer specification

^c^ Mean absolute error (MAE)

^d^ Root mean square error (RMSE)

^e^ cosine distance (d_cos)

**S2.0 Results**

**S2.1 Analysis of trajectory 4**

The most pronounced changes in RMSD and PCA were observed in trajectory **4**, a 4 μs MD simulation initiated from an AF-generated structure (**Table 1**) in which W580 adopts an outward-facing conformation and the PCASP domain begins in an active state. As shown in **Figure 1d**, the RMSD profile of trajectory **4** indicates instability, consistent with an ongoing conformational transition during the MD simulation. Detailed analysis reveals a key structural event with a conformational flip of the aromatic ring of residue W580 involving transitions in the χ₁ and χ₂ dihedral angles (**Figure 2 a, b**). Initially, the χ₁ angle remains stable around −170°, but shifts to −70° at ~800 ns, it and, reverts to −170° by ~1500 ns. During this interval, the χ₂ angle fluctuates around 100°. However, at ~2300 ns, a pronounced ring flip occurs, as χ₂ shifts from 80° to −100°. Following this transition, both angles stabilize, and the system adopts an inward-facing W580 conformation (**Figure 2c, d**).

Visual inspection of the PC1/PC2 projections (**Figure 1**) highlights the conformational diversity of trajectory **4**, in which up to four major structural clusters can be distinguished. To further characterize heterogeneity and cooperative motions within the MALT1(PCASP-Ig3)_339–719_ ensembles in trajectory **4** captured by PC1 and PC2, we visualized the corresponding PCA loadings by superposing four representative MD structures that reflect the structural variations along each principal component (**Figure 2c, d**). The transition from an outward- to inward-facing conformation of W580 in MALT1(PCASP-Ig3)_339–719_ is shown in **Figure 2d**. The flip of W580 occurs through a refolding process that involves both the Ig3 and PCASP domains, which move semi-independently, consistent with our previous findings ^1^ and as reflected by the continued RMSD changes (**Figure 1d**). A particularly intriguing result from the MD simulations was that the W580 flip seems to be coupled with the rearrangement of the active site in the PCASP domain in loop 3.

The system, which began in the active conformation, transitioned into the inactive state similar to that observed in trajectory **1** and illustrated in **Figure 2c, d**. This transition is driven by coordinated movements of loop 2 and loop 3.

A distinct two-site flip of loop 2 (cyan in **Figure 2d**) across the β3 hairpin loop (residues 416–425, yellow) occurs at approximately 2.5 µs in trajectory **4**, placing loop 2 in a position similar to that seen in the inactive starting structure of trajectory **1**. This repositioning of loop 2 subsequently displaces loop 3 to the top of the MALT1 active site (**Figure 2c**).

This rearrangement is most clearly tracked through a set of conformational “fingerprints” described by Zhang et al. ^2^, involving residues C464, R465, E549, and Q494, identified through comparisons of crystal structures of MALT1bound to an allosteric inhibitor (PDB ID: 6F7I) or a peptidic inhibitor (PDB ID:3V4O).

Here, trajectory **4** reveals that a distinctive feature of the active conformation like the presence of a short “***Elbow Loop”*** ^2,3^ (residues 491–498) within loop 3 (residues 490–509) (**Figure 2c** shown in red and **2d** leftmost structure) is absent in the inactive state (**Figure 2c** shown in blue and **2d** rightmost structure). In the inactive state, loop 3 folds over the catalytic dyad (C464–H415), effectively blocking substrate access to the active site (**Figure 2c** shown in blue). As a consequence of this rearrangement, we observed significant moves, up to 10 Å for Q494 and up to 6 Å for R465, from the active to the inactive conformations of PCASP, both of which are closely positioned to E549 in the active conformation.

**S2.2 Analysis of trajectory 3**

Similar to trajectory **4**, trajectory **3,** initiated from the outward-facing W580 conformations with the PCASP domain the inactive state, shows a dominant tendency for the aromatic ring of W580 to flip from an outward- to an inward-facing conformation. This is reflected by unstable RMSD variation (**Figure 1c**) though less pronounced than in trajectory **4** (**Figure 1d**) and by the conformational diversity observed in the PC1/PC2 projections (**Figure 1c**). As in trajectory **4**, up to four major structural clusters can be distinguished in the PC1/PC2 plane for trajectory **3**. PCA loadings further for trajectory **3** illustrate the transition of W580 toward an inward-facing conformation in MALT1(PCASP-Ig3)_339–719_, although the positions of loop 2 and loop 3 remain characteristic of the inactive state (**Figure 1c and Figure S1aB**).

These results align with our previous free energy calculations ^1^, which indicated that the inward-facing W580 conformation is energetically preferred by ~0.67 ± 0.2 kcal/mol lower in energy compared to the outward conformation.

**S2.3 Analysis of trajectory 1**

In contrast to trajectories **4** and **3**, RMSD analysis of trajectory **1** for MALT1(PCASP-Ig3)_339–719_ indicates structural stability after 0.8μs MD simulation, with no significant deviations throughout the 3 µs simulation (**Figure 1a**). This behaviour is consistent with the PCA analysis. The conformational evolution in trajectory **1** captured by PC1 and PC2 reveals a small number of low-population states early in the simulation (up to ~0.8 µs; structures coloured blue) taking place in an equilibration period of MD simulation, after which the system transitions into a single dominant cluster (**Figure 1a** purple/red) that persists throughout the remainder of the trajectory. The system, initiated with the inward-facing W580 conformation, remains in the inactive form of the PCASP domain of MALT1(PCASP-Ig3)_339–719_ under low-salt conditions (60 mM NaCl) (**Figure S1aA**).

**S2.4 Analysis of trajectory 2**

Finally, we investigated whether a spontaneous transition from the active PCASP conformation to the inactive, inward-facing W580 conformation could be captured in longer MD simulations under low-salt conditions (60 mM NaCl). To address this, we performed an additional 6μs MD simulation (trajectory **2**), starting from the AF-generated structure (**Table 1**) with the PCASP domain in the active conformation. This stands in contrast to trajectory **1**, which began with PCASP in the inactive state, although both simulations were based on an inward-facing W580 conformation.

RMSD analysis of the MALT1(PCASP-Ig3)_339–719_ trajectory **2** was calculated over backbone atoms excluding flexible loop regions, indicating an overall high structural stability with no major deviations throughout the 6 µs simulation (**Figure 1b**, dark red and blue curves). In contrast, when flexible loop regions are included, a transition to a higher RMSD plateau is observed at approximately 2.5 µs (**Figure 1b**, light pink and grey curves). This behaviour is consistent with the PCA results in which the PC1/PC2 projections for trajectory **2** correspond to two dominant clusters (purple/red) that persist throughout the remainder of the simulation.

Supplementary **Figure S1** shows superpositions of 30 structures sampled from opposite ends of the structural span defined by the PC1 modes for all conformational ensembles. Visual inspection (**Figure S1aC**) reveals two key features: (i) the inward-facing orientation of the W580 aromatic ring is maintained throughout trajectory **2**, consistent with the behaviour observed in trajectory **1**; and (ii) loop 2 flips around the protruding β-sheet spanning residues 416–426, leading to a transition of the PCASP domain from an active to an inactive conformation.

**S2.5 Analysis of trajectories 8, 9**

To further probe the behaviour of the PCASP domain under high-salt conditions, we carried out a 4 μs simulation (trajectory **9**), using the same AF-generated model as in trajectory **4**, i.e. with W580 in an outward-facing conformation and an active PCASP domain. RMSD analysis (**Figure** **4c**) shows that trajectory **9** remains close to the starting structure. Consistently, W580 retains its outward orientation, and the PCASP active site remains in the active state. The PC1/PC2 analysis likewise shows a single, well-defined conformational family (**Figure** **4c1, c2**), in contrast to the broad distribution observed for trajectory **4** (**Figure** **4d1, d2**) after the equilibration period (~0.3 μs).

A striking difference emerges when the 8 μs combined trajectories (trajectories **9** + **4**) is subjected to conformational clustering (see Methods). As shown in **Figure 4f**, trajectory **9** occupies a single, well-populated local minimum, whereas trajectory **4** displays no stable clusters, consistent with its higher conformational heterogeneity. The two trajectories populate non-overlapping regions of conformational space.

We next performed a 3 μs simulation (trajectory **8**) starting from the same AF-generated structure used for trajectory **2**, in which W580 is inward-facing and the PCASP domain adopts an active conformation. Unlike trajectories **4** and **9**, which show pronounced differences in structural drift, the RMSD profiles of trajectories **8** and **2** are more similar and less conclusive. It seems that RMSD analysis of trajectory **8** (**Figure** **4a**) indicates a stable structure with no major deviations from the starting conformation. Structural inspection confirms that W580 remains inward-facing and that the active site of the PCASP domain is preserved throughout the simulation.

PC1/PC2 analysis shows two well-defined conformational families in trajectories **8** and this in also found in trajectory **2** (**Figure** **4a1, a2** and **4b1, b2**). However, trajectory **2** undergoes a transition from the active to the inactive PCASP conformation, whereas this transition is not observed in trajectory **8**. Clustering of the combined trajectories **8** + **2** (**Figure** **4e**) reveals several local minima, again indicating conformational heterogeneity. The clusters populated by the two trajectories are non-overlapping.

An unexpected and notable result is that trajectory **9** is more structurally stable than trajectory **8** under high-salt conditions, as demonstrated by clustering of the combined trajectories (**Figure** **4g**). Trajectory **9** occupies a single, well-populated local minimum, while trajectory **8** populates several small, unstable clusters. No overlap is observed between their respective cluster distributions.

**S2.6 Methyl Group Dynamics as Probes of Conformational Stability: Agreement Between MD and NMR**

In our previous study on the apo form of human MALT1(PCASP-Ig3)_339–719_ ^4^ we reported nearly complete ^1^H /^13^C methyl resonance assignments for Ile, Leu, and Val residues. These findings provide a solid foundation for further investigation of both backbone and side-chain dynamics in the monomeric form of MALT1.

The longitudinal relaxation rate (R_1_) and cross-correlated relaxation rate (Г_2_), both measured at 800 MHz, are shown in light blue solid brackets in **Figure S5**. For methyl groups, R_1_R₁ values span ~1.0–5.0 s⁻¹, while Γ_2_ values range from ~5.0–25.0 s⁻¹, reflecting a broad distribution of internal correlation times (τₑ) and order parameters (S²).

Additionally, the ^13^C relaxation parameters for ^13^C_d2_ for methyl group of Leu amino acid should be interpreted with caution due to the possible formation of so-called AB spin systems with ^13^C_g_, which can occur when the chemical shift difference is smaller than ~350 Hz (≈10 × ^1^J(C_d2_C_g_)).

Closer inspection of R_1_ relaxation reveals clear amino acid–specific differences. Ile methyl groups display the narrowest distribution, with values clustered around 1.0 s⁻¹ (**Figure S5e**), despite being distributed across diverse structural elements of MALT1(PCASP-Ig3)_339–719_. By contrast, Val and Leu methyl groups show higher average R_1_ values (~2.5 s⁻¹) and greater variability, including slight sequence-dependent effects (**Figure S53a, c**). These trends suggest that τₑ is governed in general more strongly by amino acid type than by sequence context.

Analysis of Γ_2_ profiles, which reflect the order parameter S², clearly highlights the heterogeneity in dynamic behaviour across all three amino acid types (**Figure S53b, d and f**), emphasizing the complexity of methyl side-chain motion in MALT1. This variability enables the use of Г_2_ as a benchmark for evaluating theoretically back-calculated parameters and comparing them across trajectories ensembles **1**, 8 and **9**.

Inspection of **Figure** **S5** indicates that, overall, trajectory **1** aligns closely with the experimental data, falling in general within the range of experimental uncertainties for both R_1_ and Г_2_. We consider this result important from a methodological point of view, as it indicates that free MD dynamics reproduces seemingly correctly the dynamics of methyl side chains for Val, Leu and Ile residues. This finding is particularly notable given that force field parameters for methyl groups are still under investigation. Moreover, the result expected to be if methyl dynamics in MALT1(PCASP-Ig3)_339–719_ are consistent with the backbone dynamics observed in ensemble of trajectory **1.**

It is essential to emphasize that the other trajectories, corresponding to conformational ensemble **8, 9** obtained under higher salt conditions and exhibiting distinct active-site conformations but with different **W580** aromatic ring orientations, also align well with the experimental data (**Figure S5**). A striking example is the nearly identical R_1_ and Γ_2_ relaxation parameters for **Ile501** (**Figure S5e, f**) across ensembles (**1, 8** and **9**), despite the substantial repositioning of this residue in the active versus inactive conformation. This suggests that the loop 3 conformational transition in the PCASP domain has minimal impact on the fast methyl group dynamics.

**S2.7 Relaxation Analysis Across Hydrophobic Clusters**

To identify methyl groups most affected by interdomain interactions between Ig3 and PCASP, we sorted Val, Leu, and Ile methyl’s into four distinct hydrophobic clusters (Cl1–Cl4, **Figure** **S6**). These clusters are: Cl1 (yellow) within the Ig3 domain, Cl2 (violet) at the Ig3–PCASP interface, and Cl3 and Cl4 (green and red, respectively) positioned on opposite sides of the PCASP β-sheet.

For Cl1, which is distant from the major conformational differences between ensembles **1, 8** and **9**, the deviations between experimental and back-calculated R_1_ and Γ_2_ values are expected to be minimal (**Figure S6d1, d2**) conformational changes exert little influence on fast dynamics within these clusters.

Similarly, for surface-exposed methyl groups (**Figure S6e1, e2**), trajectories ensembles (**1, 8** and **9**) exhibit strong agreement with experimental data, indicating that salt concentration does not significantly affect the fast dynamics of these methyls.

***Cl2: A Critical Cluster at the Ig3–PCASP Interface***

The Cl2 cluster is particularly significant as it is located at the Ig3–PCASP interface, near the active site. Previous studies ^1^ suggest that Cl2 plays a crucial role in MALT1(PCASP-Ig3)_339–719_ interactions with allosteric ligand. Nevertheless, the relaxation parameters R_1_ and Γ_2_ of the Cl2 methyl groups (**Figures S6c1, c2**) reveal that the back-calculated values for trajectories (**1**, **8** and **9**) similar and closely match the experimental data.

**S2.8 Backbone Dynamics of MALT1(PCASP–Ig3)_339-719_ Conformation Ensembles**

To investigate residue-level dynamics in the MALT1(PCASP–Ig3)_339-719_ ensembles, we applied two complementary approaches: analysis of Cα root-mean-square fluctuations (RMSF) and evaluation of the NH-vector autocorrelation function, acf(t).
First, we analysed per-residue RMSF profiles to assess regions of structural flexibility across the MD trajectories of MALT1(PCASP–Ig3)_339-719_. The averaged RMSF values for trajectories **1**, **8**, **9,** and **11** are shown in **Figure 7a**. Overall, residues 560–720 remained relatively stable in all simulations, with fluctuations below ~2.5 Å. The major differences between trajectories were localized to loop 2, loop 3, and the linker region connecting the PCASP and Ig3 domains (loop 6).

In trajectory **1**, which represents the inactive ensemble, loop 3 exhibits the largest motions, with RMSF values exceeding 8 Å, whereas loop 2 remains highly ordered (RMSF < 2.5 Å). The pronounced flexibility of loop 3 may reflect a prerequisite dynamic state that enables its eventual transition toward the active conformation.

In trajectory **9**, corresponding to a stabilized active conformation with W580 rotated outward, loop 2 shows the greatest flexibility (RMSF up to ~6 Å), consistent with allosteric pressure toward the inactive loop 2 position despite the overall rigidity imposed by high-salt conditions.

By contrast, trajectory **8**, active-like but with W580 already oriented inward, exhibits intermediate fluctuations across all loops, suggesting that this ensemble samples multiple local minima within the active-state basin.

Interestingly, loop 1, located near the active site, remains relatively stable in all simulations (RMSF < 2.5 Å), with only a slight increase in flexibility observed in loop 5 (RMSF ≈ 3 Å).This contrasts with the RMSF values obtained for conformational ensemble trajectory **11**, in which increased flexibility is observed in the α5 helix and loop 5 region (RMSF ≈ 8-10 Å), indicating substantial disordering of this region of MALT1.

In contrast to RMSF analysis, which reports the average spatial displacement of Cα atoms across the simulated conformational ensemble, the NH-vector autocorrelation function, acf(t), provides time-resolved information on backbone angular stability. Specifically, acf(t) describes how long the orientation of each NH bond vector remains correlated, thereby revealing the persistence of local structural order over different timescales.

To assess these backbone-motion timescales, we calculated the NH-vector autocorrelation values for every residue of MALT1(PCASP–Ig3)_339-719_ at four representative time points: t = 0.1, 1.0, 10, and 100 ns. The resulting acf(t) profiles, extracted from the corresponding MD segments, are shown in **Figure 7** for trajectory **1** (panel b), trajectory **8** (panel c), and trajectory **9** (panel d).

As expected, all trajectories show minimal time-dependent changes in acf(t) for residues 340–460, 570–690, and 700–720, with values remaining in the range of 1.0–0.8. This indicates that the NH vectors in these regions are highly stable and dominated by fast picosecond-scale motions. In contrast, the largest deviations and the strongest time-dependent decay in acf(t) are observed in loops 1–6, with loops 2, 3, and 6 reaching values as low as ~0.2 at the 100 ns time point. This pronounced decay suggests the presence of slower motional components within the 500 ns window from which the autocorrelation functions were calculated. Because acf(t) does not reach a plateau for these loop regions, it is likely that additional slow dynamical processes occur on timescales longer than 500 ns.

Together, the RMSF and autocorrelation analysis show that the structured core of MALT1(PCASP–Ig3)_339-719_ remains highly stable across all ensembles, whereas backbone dynamics are concentrated in the loop regions. loop 2 and loop 3 exhibit the strongest conformation-dependent motions, with loop 3 most flexible in the inactive ensemble and loop 2 most flexible in the active ensemble, reflecting their distinct roles in the inactive-to-active transition. Autocorrelation profiles further reveal that these loops undergo slow, long-timescale motions that extend beyond the 500 ns analysis window, whereas the remaining structured regions are dominated by fast picosecond dynamics. Overall, the data indicate that the loop dynamics, rather than the domain cores, define the conformational plasticity of MALT1 and govern its transitions between inactive and active states.

**S3 Methods**

## **S3.1 Expression of isotope-labelled MALT1(PCASP-Ig3)_339–719_ and preparation of NMR samples**

Expression, purification and NMR sample preparation was fully explained in ^1^. Shortly the DNA sequence encoding for the PCASP and Ig3 domains of human MALT1, corresponding to residues 339–719 and a C-terminal His6-tag was cloned into the expression vector pET21b (Novagen). MALT1(PCASP-Ig3)_339–719_ was transformed into *Escherichia coli* strain T7 express competent cells (NEB) and expressed in different isotopic labelling combinations in ^1^/^2^H, ^15^N, ^12^/^13^C-labelled M9 medium. One hour prior to induction, precursors were added to the growth medium as previously described ^5^. For precursors, 70 mg/L alpha-ketobutyric acid, sodium salt (^13^C4, 98%, 3,3-^2^H, 98%) and 120 mg/L alpha-ketoisovaleric acid, sodium salt (1,2,3,4-^13^C4,99%, 3, 4, 4, 4, -^2^H 97%) (CIL, Andover, MA) were used. Cells were harvested and lysed using ultra-sonicator followed by centrifugation to remove cell debris. The supernatant containing MALT1 was purified by Ni^2+^ Sepharose 6 Fast Flow (Cytiva). A Q-Sepharose HP column (Cytiva) was used to separate monomeric MALT1(PCASP-Ig3)_339–719_ from the dimer form. A final size exclusion chromatography (SEC) step was performed using a HiLoad 16/600 Superdex 200 prep grade column (Cytiva), with running buffer 20mM HEPES 7.4, 50mM NaCl, 1mM DTT. The final monomer MALT1(PCASP-Ig3)_339–719_ protein sample was subsequently exchanged to a buffer (10 mM Tris 7.6, 50mM NaCl, 2mM TCEP, 0.002% NaN_3_, 10% D_2_O) using gravity flow PD10 desalting column (Cytiva). The purified monomeric MALT1(PCASP-Ig3)_339–719_-His protein was concentrated to at least 0.3-0.5 mM for NMR data acquisition.

## **S3.2 NMR relaxation experiments and data processing**

All spectra were processed using either the mddnmr ^6^ and the NMRPipe ^7^ softwares at the NMRbox server (<https://nmrbox.nmrhub.org/>) ^8^ or with the TopSpin 4.3.0 (Bruker, Billerica, MA, USA) and analysed using CcpNmr2.4.2^9^ and Dynamics Center 2.8.4 (Bruker, Billerica, MA, USA). Molecular graphics were prepared with the use of Chimera 1.16 and ChemeraX ^10,11^

**S3.2.1 Determination of ^1^H-^15^N CSA/dipole cross correlation (η_xy_) relaxation**

Backbone ^1^H-^15^N CSA/dipole cross-correlated relaxation rates (ηₓᵧ) were measured at 900 MHz using a modified version^12^ of the previously reported pulse sequences ^13-15^. Experiments were performed using NS=40 on a time domain grid of 1 K x 100 complex points with spectral width/acquisition time of 16 ppm/71 ms for ^1^H and 40 ppm/27 ms for ^15^N dimensions with D1 = 1s and at a constant-time delay of T = 0.03s. η_xy_ values were determined from a series of 8 relaxation delays: 0.000, 0.002, 0.005, 0.009, 0.014, 0.023, 0.030, 0.044. Carrier positions: ^1^H, H_2_O frequency (4.7 ppm); ^13^C, 95 ppm; ^15^N, 118.0 ppm. Mirror image linear prediction was used for constant-time ^15^N sampling.

**S3.2.2 Methyl ^13^C–¹H_3_ relaxation experiments**

Relaxation measurements for methyl ^13^C-^1^H_3_ groups ^16,17^ were performed at 800MHz using an interleave pseudo 3D spectral acquisition. Experimental longitudinal relaxation rates (R_1_) of ^13^C-^1^H_3_ groups were obtained as previously described ^18,19^ by applying mono-exponential fitting to cross peak intensities across a series of 12 two-dimensional correlation spectra. These spectra were recorded with T1 relaxation delays of 0.01, 0.04, 0.08, 0.13, 0.20, 0.29, 0.41, 0.57, 0.69, 0.99, 1.39 to 2.00 s. The number of transients was set to 16.

The dipolar CH, CH cross-correlation contribution to R_2_ (named in this study as Γ_2_) for ^13^C-^1^H_3_ groups was measured as previously described ^17,18^ , using a constant time period of 28.6ms and 14 evolution delays (Δ) of 0.01, 0.6, 1.2, 1.8, 2.4, 3.0, 3.6, 4.2, 4.8, 5.6, 6.4, 7.2, 8.0 to 9.2ms. The number of transients was set to 32.

All parameters in experiments for measuring R_1_ and Γ_2_ relaxation on ^13^C-^1^H_3_ groups, were set as previously described ^18,19^. The ^1^H and ^13^C carrier frequencies were set to the water resonance at 4.7 and 16.5 ppm, respectively. The spectral width (SW) for ¹H was 12 ppm over 1024 complex points, while for ¹³C it was 16 ppm over 80 complex points. The inter-scan delay was set to 1 s.

Processing of R_1_ and Γ_2_ spectral datasets was performed using TopSpin4.3.0 software (Bruker) and analysed with the Mathematica software package (Wolfram Research Inc.) as previously described ^18,19^.

**S3.3 Molecular Dynamic simulation**

**S3.3.1 Starting structures for MD-simulations**

Both the experimental X-ray structures (PDB 3V55) and the predicted structure from AlphaFold [<https://www.nature.com/articles/s41586-024-07487-w>] were used as starting structures for the MALT1 protein in solution (Figure S9). Structural modifications and modelling of the missing loops regions (pdb id 3v55) and missing α-helix region (pdb id 9mkd) of the crystal structure were performed with PDBFixer ^20^. However, the primary focus of this work was not only the initial conformations of MALT1 but also the solution properties, such as ionic strength (IS), while pH 7.6 and temperature of 298 K remained constant for all MD runs.

According to AF, MALT1(PCASP-Ig3)_339_–_719_ adopts two major structural states distinguished by the orientation of the aromatic ring of residue W580, which is positioned either within or outside the pocket between the PCASP and Ig3 domains. Throughout the remaining part of this text, these conformations will be referred to as inward- or outward-facing W580 conformations, corresponding starting structures I, II and III, IV, respectively (**Table 1**). In addition, the PCASP domain may adopt either an inactive or an active conformation, corresponding to structures I, III and II, IV, respectively (Table 1). The inactive state resembles the previously determined crystal structure (PDB ID: 3V55), whereas the active state corresponds to the conformation predicted by AF ^1^ and resembles the conformations found in crystal structures bound to substrate-mimicking ligands (PDB ID: 3V4O).

The combinations of starting conformations (SCs) of MALT1 and ions concentrations used in the simulations are presented in **Table 1** and **Figure S9, 10**.

**S3.3.2 The Protocol used in MD Simulations**

This study investigates intramolecular electrostatic interactions and their impact on the time-resolved conformational dynamics of MALT1 monomers, referred to as “4D structural biology” to denote the time-dependent three-dimensional ensembles obtained from molecular dynamics (MD) simulations ^21^.

A well-recognized limitation of non-polarizable all-atom force fields is the accurate representation of charged groups ^22^. To address this issue, several studies have proposed charge scaling schemes for both proteins and ions ^23-25^. Although the TIP3P water model remains the *de facto* standard due to its broad compatibility with biomolecular force fields, it has been argued to insufficiently capture the complexity of water–ion and water–protein electrostatic interactions ^26,27^. Accordingly, careful consideration of simulation protocols was a prerequisite for this study.

Because intramolecular electrostatic interactions are most pronounced under conditions of low ionic strength, owing to reduced electrostatic shielding, we systematically evaluated and benchmarked alternative MD protocols against experimental NMR relaxation data collected under low-salt conditions.

Two molecular dynamics protocols were systematically evaluated: (1) CHARMM36 force field with CUFIX corrections and (2) polarizable AMOEBA model, which explicitly accounts for polarization effects in proteins, ions, and, most critically, water molecules.

**S3.3.3 MD Simulations Using the Classical Non-Polarizable CHARMM36 Force Field**

MD simulations were performed using GROMACS version 2023.1 ^28^ with the all-atom force field charmm36-mar2019_cufix.ff ^29-31^, including a refinement of Lennard-Jones parameters (CUFIX) ^32^. Both the protein and the TIP3P water model were treated according to recommended parameters.

The protein was placed at the centre of a periodic cubic box (104 Å), with 55 Na^+^ and 41 Cl^–^ ions added to match the 60 mM ionic strength. The NMR experiments were performed in buffer containing 50 mM NaCl. In MD simulations, the low ionic strength was set to ~60 mM by explicitly adding ions, which accounts for both the nominal salt concentration and additional ionic strength contributions from buffer components and residual ions present in experimental conditions. 415 Na^+^ and 401 Cl^–^ ions added to match the 500 mM ionic strength and maintain electro-neutrality, given the protein’s total charge of -14. For MD with trisodium citrate (3Na^+^ and C_6_H_5_O_7_³⁻) ions, an additional parameterization of citrate ions with charge -3 was performed in the CGenFF software package ^33,34^. To achieve target sodium ion (Na⁺) concentrations of 500 mM and 1500 mM, corresponding amounts of 416 and 1355 Na⁺ ions, along with 134 and 447 citrate (C₆H₅O₇³⁻) ions, were added respectively. Since residues charges were calculated according to pH 7.6, all histidine residues remained neutral. A cutoff of 12 Å was applied for both long-range electrostatic and van der Waals interactions.

To ensure a well-defined starting structure, energy minimization was performed, achieving convergence at a maximum force below 1000 kJ/mol/nm per atom.

Equilibration of the system was performed at 298 K through two restrained phases. Each phase lasted 100 ps and employed positional restraints on protein’s heavy atoms. The initial phase used the NVT ensemble (constant number of particles, volume, and temperature) to stabilize the system temperature. The second phase used the NPT ensemble (constant number of particles, pressure, and temperature) to allow the system density to adjust. Following this, all restraints were removed, and a free production molecular dynamics simulation was carried out in the NPT ensemble for 2500 ns until the system reached stability, indicated by a plateau in root mean square deviation (RMSD) values.

A modified Berendsen-type (V-rescale) thermostat and a Parrinello-Rahman barostat were employed. Hydrogen-containing covalent bonds were constrained using the Links algorithm with a 2 fs timesteps. Following equilibration, MD simulations were continued as a production run for 500 ns under the same conditions. System stability was assessed using standard GROMACS tools ^28^ including control of the temperature, pressure, energy, secondary structure, the box border, and RMSD.

**S3.3.4 Molecular Dynamics Simulations Using the Polarizable AMOEBA Force Field**

Electrostatic interactions play a crucial role at low ionic strength due to reduced shielding. Therefore, an MD simulation was performed in Tinker-9 using the polarizable AMOEBA force field to assess whether the classical non-polarizable force field sufficiently agrees or not with experimental results.

The protein residue charges were calculated at pH 7.6, in the same way as for GROMACS in the previous section, with all histidine residues remaining neutral. The ionic strength was set to 60 mM, accounting for both buffer and salt concentrations.

MD simulations were performed using Tinker-9 [Tinker9: Next Generation of Tinker with GPU Support. Zhi Wang, Jay W. Ponder, 2021, https://github.com/TinkerTools/tinker9] with the all-atom polarizable force field AMOEBABIO18 ^35^. The protein was placed at the centre of a periodic cubic box (103.9375 Å), with 55 Na^+^ and 41 Cl^-^ ions added to match the desired ionic strength and maintain electro-neutrality, given the protein’s total charge of -14. Molecular dynamics simulations were performed using the RESPA integrator with a 2 fs outer time step and a preconditioned conjugate gradient polarization solver (with a 10^-5^ convergence threshold). Periodic boundary conditions (PBCs) were applied using the Smooth Particle Mesh Ewald (SPME) method, with a 120 × 120 × 120 Å grid. The Ewald-cut off was set to 7 Å, while van der Waals and electrostatic charge-charge cut offs were 12 Å. Mutual dipole polarization was applied to iterate induced dipoles to self-consistency, with a convergence cutoff of 10^−5^ Debye.

Before the simulation, the protein structure underwent energy minimization to optimize starting structure geometry and solvent orientation. Convergence was achieved at a maximum force below 0.01 kcal/mol/Å per atom.

Equilibration was performed in two phases. First, NVT ensemble was applied 100 ps equilibration. The system was heated to 298 K until the temperature plateaued. Second, NPT ensemble 100 ps was used for the continued equilibration until pressure and density stabilized. A Bussi-Parrinello stochastic thermostat and a Monte Carlo barostat were used. The RESPA integrator was applied. Following equilibration, MD simulations continued as a production run for 3000 ns under the same conditions. System stability was monitored using standard Tinker9 tools, tracking temperature, pressure, energy and periodicity.

Finally, we evaluated whether a polarizable force field improves accuracy under low-ionic-strength (55 Na^+^ and 41 Cl^-^) conditions. Using starting structure I, we compared MD simulations performed with the CHARMM36 + CUFIX force field (trajectory **1**) and the polarizable AMOEBA force field (trajectory **12**). Back-calculated R_1_ and η_xy_ relaxation data (**Figure** **S7**) showed no improvement with AMOEBA (**Table S2**).

One possible explanation for these differences is that MALT1 is not an intrinsically disordered protein, for which polarizable force fields such as AMOEBA are often particularly beneficial ^36^, and that in this system not only electrostatic interactions but also van der Waals and bonded interactions play a significant role in shaping the conformational landscape. In addition, for a protein of this size, CHARMM provides a substantial computational advantage over AMOEBA, enabling longer simulations and more extensive sampling.

Therefore, the CHARMM19 + CUFIX force field provides sufficient accuracy for studying MALT1 conformational dynamics and was adopted for all subsequent simulations.

**S3.4 MD Trajectory Analysis: Alignment, RMSD Calculation, and Back-Calculation of NMR Relaxation Parameters**

**S3.4.1** **Alignment, RMSD, PCA**

To assess the quality of the reconstructed trajectories, we evaluated several ensemble properties that describe global structural behaviour, including root-mean-square deviation (RMSD) and Principal Component Analysis (PCA). RMSD is the primary metric used to quantify structural variation within the ensemble. As shown in **Figure 1**, RMSD fluctuations were analysed in four ways using the Cα backbone atoms of MALT1(PCASP-Ig3)_339–719_​. RMSD was calculated relative to either the initial structure or the representative structure of the most populated cluster. These analyses were performed for (i) selected residue ranges (5–130, 147–154, 172–225, and 235–380) excluding flexible loop regions, and (ii) all residues except the N- and C-terminal segments (residues 1–4 and 381–388).

Structural alignments and subsequent RMSD calculations were performed using two distinct sets of backbone heavy atoms 1: [342-717] and 2: [342-467, 484-491, 509-562, 572-717], excluding the mobile N- and C-terminal residues (338-341and 718-725) in both cases. In the second set structures were aligned excluding the mobile inter-domain linker (563-571) and flexible loops (468-483 and 492-508), followed by an RMSD calculation for both sets of backbone heavy atoms. All RMSD values were computed using the GROMACS software package.

PCA is a dimensionality-reduction technique essential for visualizing and characterizing the dominant motions in molecular ensembles, providing a complementary perspective to RMSD for describing global dynamics. To assess whether our models capture these key dynamical features, we analysed the first two principal components (PC1 and PC2) for trajectories **1**, **2**, **3** and **4**. PCA was performed using two residue selections intervals: residues 5–380 (**Figure** **1**, left PCA panels) and residues 5–130, 147–154, 172–225, and 235–380 (**Figure** **1**, right PCA panels).

Principal component analysis (PCA) was performed on Cartesian coordinates for all molecular dynamics (MD) trajectories using the GROMACS software package. The analysis was conducted on the backbone heavy-atoms, analogous to those used for the RMSD calculation. For each trajectory, 30 eigenvalues were calculated. The data for the first three eigenvalues corresponding to the first three principal components (PCi) are presented in **Table S1.**

RMSF values were calculated for Cα atoms after centre-of-mass alignment of all trajectory frames and was computed using the standard definition ^37^.

**S3.4.2** **Individual MD trajectory analysis with back-calculation of theoretical** **^15^N and ^13^C relaxation parameters**

The back-calculation of NMR relaxation parameters followed the method described in our earlier publications ^12,19^, using the final 500 ns of the trajectory for calculating the correlation function with maximal time of 7×τ_c_, with an experimental overall correlation tumbling of τ_c_ =27ns. MD trajectory regions were analysed by back-calculation NMR spin-relaxation parameters, using a bootstrapping procedure to estimate parameters dispersion, as previously described ^19^. Each 500 ns MD segment was selected to be several times longer than the maximum duration of the autocorrelation function acf(t), specifically exceeding 7×τ_c_, to ensure proper averaging of acf(t) values and effective application of the moving block bootstrap method ^19^. For each MD segment, the analysis began by aligning all protein frames to the mean structure, using the heavy atoms of rigid backbone residues.

The backbone ^1^H-^15^N vector extraction and approximation of autocorrelation function acf(t) to a multi-exponential decay

$\mathrm{acf}\left( t \right)\text{=A}_{0}+\sum_{\text{j=}1}^{m} A_{j}e^{{-t}/{\tau_{j}}}$ (1)

with the best-fit parameters A_0,_ A_j,_ τ_j_ and the subsequent spectral density function J(ω) calculations were utilized as previously described ^12,19^, using homebuilt scripts in “Mathematica” software package [Wolfram Research] and the MD Analysis external library [mdanalysis.org]. The resulting acf(t) values could be calculated with respect to t values (0.1ns, 1ns, 10ns, 100ns) presenting the time scale of the motions (**Figure S8**).

Back-calculation of classical NMR ^15^N relaxation parameters η_xy_, R_1,_ R_2_ and NOE as a function of J(ω) were also performed as following:

$R_{1}=\frac{1}{\text{10}}\left( \frac{\mu_{0}h{\gamma_{H}\gamma}_{N}}{8\pi^{2}r_{\text{NH}}^{3}} \right)^{2}\left[ J\left( \omega_{H}-\omega_{N} \right)+\text{3J}\left( \omega_{N} \right)+\text{6J}\left( \omega_{H}{\text{+}\text{ω}}_{N} \right) \right]+\frac{2\omega_{N}^{2}\text{Δσ}^{2}}{15}J\left( \omega_{N} \right)$ (2)

$NOE=1+\frac{\gamma_{H}^{3}\gamma_{N}}{10R_{1}}\left( \frac{\mu_{0}h}{8\pi^{2}r_{NH}^{3}} \right)^{2}\left[ \text{6J}\left( \omega_{H}{\text{+}\text{ω}}_{N} \right)-J\left( \omega_{H}-\omega_{N} \right) \right]$ (3)

$R_{2}=\frac{1}{\text{20}}\left( \frac{\mu_{0}h{\gamma_{H}\gamma}_{N}}{8\pi^{2}r_{\text{NH}}^{3}} \right)^{2}\left[ 4J\left( 0 \right)+\text{3J}\left( \omega_{N} \right)+J\left( \omega_{H}-\omega_{N} \right)+6J\left( \omega_{H} \right)+6J\left( \omega_{H}{\text{+}\text{ω}}_{N} \right) \right]+\frac{\omega_{N}^{2}\text{Δσ}^{2}}{45}\left[ 4J\left( \omega_{0} \right)+3J\left( \omega_{N} \right) \right]$ (4)

Cross-correlated relaxation rates (η_xy_) arising from interference between the ^1^H–^15^N dipolar interaction and the ^15^N chemical shift anisotropy (CSA) were determined using described previously ^14,15,38^.

$\eta_{xy}=-\frac{1}{15}\left( \frac{\mu_{0}{h\gamma}_{H}\gamma_{N}^{2}B_{o}\Delta\sigma P_{2}\left( cos(\theta) \right)}{8\pi^{2}r_{\mathrm{NH}}^{3}} \right)\left[ 4J\left( 0 \right)+3J\left( \omega_{N} \right) \right]$ (5)

Where the spectral density function was:

$J\left( \omega\right)=\frac{A_{0}\tau_{c}}{1+\left( \text{ωτ}_{c} \right)^{2}}+\sum_{\text{j=}1}^{m} \frac{A_{j}\tau_{j}'}{1+\left( \text{ωτ}_{j}' \right)^{2}}$ (6)

Where $\tau_{j}\text{'=τ}_{c}{\tau_{j}}/{\tau_{c}}\text{+τ}_{j}$ and $\tau_{c}$ is the experimental rotation correlation time, $\mu_{o}$ is the vacuum permeability; h is Planck's constant; $Y_{H}$ and $Y_{N}$ are the gyromagnetic ratios of ^1^H and ^15^N respectively; $\Delta\sigma$ is the chemical shift anisotropy (CSA) of ^15^N with Δσ = −166±9ppm ^39^; r_NH_ = 1.023±0.006 Å ^40^; ω_N_ and ω_H_ are the Larmor frequencies of ^15^N and ^1^H at 800 or 900 MHz, respectively; Bo is the static magnetic field strength; CSA tensor value with respect to the NH vector ΔσP_2_(Cos(θ)) = −145±8ppm, θ is the CSA/NH vector angle and P_2_ is the Legendre 2nd degree polynomial ^41^; J(ω) is the NH auto-correlation spectral density function.

**S3.5 Penalty functions for MD trajectory validation**

An approach was used to identify the most representative molecular dynamics (MD) trajectory that agrees well with experimental NMR relaxation data. The ranking was performed by comparing back-calculated relaxation parameters (η_xy_, NOE, R_1_, and R_2_) back-calculated from the MD trajectories with the corresponding experimental one. First, both theoretical and experimental values were normalized with respect to the corresponding mean experimental values:

$\bar{x}_{ex}=\frac{1}{n}\sum_{i=1}^{n} x_{ex,i}$

Where i is residue number, $x_{ex,i}$ is i-th experimental value of the relaxation parameter, $\bar{x}_{ex}$ is the average value of the relaxation parameter and n is the number of values used in the ranking.

$x_{ex,i}^{'}=\frac{x_{ex,i}}{\bar{x}_{ex}}$

where $x_{ex,i}^{'}$ is the new normalized experimental i-th value.

$x_{theor,i}^{'}=\frac{x_{theor,i}}{\bar{x}_{ex}}$

where $x_{theor,i}^{'}$ is back-calculated i-th value of the relaxation parameter for MD trajectory and $x_{theor,i}^{'}$ is the new normalized i-th value.

The analysis included data from amino acid residues, excluding the following regions: 340–350, 364–400, 407–419, 444–448, 455–462, 487–488, 525–538, 578–621, 631–639, 644–655, 679–692, 700–715, and the His-tag. Based on these data, the ranking was conducted according to three principal metrics: mean absolute error (MAE), root mean square error (RMSE) ^42^ and cosine distance ^43^.

$$MAE=\frac{1}{n}\sum_{i=1}^{n} \left| x_{ex,i}^{'}-x_{theor,i}^{'} \right|$$

$$RMSE=\sqrt{\frac{\sum_{i=1}^{n} \left( x_{ex,i}^{'}-x_{theor,i}^{'} \right)^{2}}{n}}$$

For the calculation of the cosine distance, the experimental parameter values were arranged into a vector $\vec{a}$ = [$x_{ex,1}^{'}, x_{ex,2}^{'}\ldots, x_{ex,n}^{'}$], while the theoretical values were arranged into a vector $\vec{b}$ = [$x_{theor,1}^{'}, x_{theor,2}^{'}\ldots, x_{theor,n}^{'}$] The cosine distance was computed using the equation (7). The data of penalty functions are presented in **Table** S**2**.

**References**

1 Wallerstein, J. *et al.* Insights into mechanisms of MALT1 allostery from NMR and AlphaFold dynamic analyses. *Commun Biol* **7** (2024). <https://doi.org:10.1038/s42003-024-06558-y>

2 Zhang, J. R., Ren, L., Wang, Y. & Fang, X. X. In silico study on identification of novel MALT1 allosteric inhibitors. *Rsc Adv* **9**, 39338-39347 (2019). <https://doi.org:10.1039/c9ra07036b>

3 Wiesmann, C. *et al.* Structural Determinants of MALT1 Protease Activity. *J Mol Biol* **419**, 4-21 (2012). <https://doi.org:10.1016/j.jmb.2012.02.018>

4 Han, X. *et al.* Assignment of IVL-Methyl side chain of the ligand-free monomeric human MALT1 paracaspase-IgL(3) domain in solution. *Biomolecular Nmr Assignments* (2022). <https://doi.org:10.1007/s12104-022-10105-3>

5 Tugarinov, V., Kanelis, V. & Kay, L. E. Isotope labeling strategies for the study of high-molecular-weight proteins by solution NMR spectroscopy. *Nat Protoc* **1**, 749-754 (2006). <https://doi.org:10.1038/nprot.2006.101>

6 Orekhov, V. & Jaravine, V. A. Analysis of non-uniformly sampled spectra with multi-dimensional decomposition. *Prog Nucl Mag Res Sp* **59**, 271-292 (2011). <https://doi.org:doi.org/10.1021/ja062146p>

7 Delaglio, F. *et al.* Nmrpipe - a Multidimensional Spectral Processing System Based on Unix Pipes. *Journal of Biomolecular Nmr* **6**, 277-293 (1995). <https://doi.org:Doi> 10.1007/Bf00197809

8 Maciejewski, M. W. *et al.* NMRbox: A Resource for Biomolecular NMR Computation. *Biophysical Journal* **112**, 1529-1534 (2017). <https://doi.org:10.1016/j.bpj.2017.03.011>

9 Vranken, W. F. *et al.* The CCPN data model for NMR spectroscopy: development of a software pipeline. *Proteins* **59**, 687-696 (2005). <https://doi.org:10.1002/prot.20449>

10 Pettersen, E. F. *et al.* UCSF chimera - A visualization system for exploratory research and analysis. *Journal of Computational Chemistry* **25**, 1605-1612 (2004). <https://doi.org:10.1002/jcc.20084>

11 Pettersen, E. F. *et al.* UCSF ChimeraX: Structure visualization for researchers, educators, and developers. *Protein Science* **30**, 70-82 (2021). <https://doi.org:10.1002/pro.3943>

12 Lesovoy, D. *et al.* Accurate Protein Dynamic Conformational Ensembles: Combining AlphaFold, MD, and Amide N(H) NMR Relaxation. *Int J Mol Sci* **26** (2025). <https://doi.org:ARTN> 8917 10.3390/ijms26188917

13 Liu, Y. Z. & Prestegard, J. H. Direct measurement of dipole-dipole/CSA cross-correlated relaxation by a constant-time experiment. *J Magn Reson* **193**, 23-31 (2008). <https://doi.org:10.1016/j.jmr.2008.03.013>

14 Tjandra, N., Szabo, A. & Bax, A. Protein backbone dynamics and N-15 chemical shift anisotropy from quantitative measurement of relaxation interference effects. *J Am Chem Soc* **118**, 6986-6991 (1996). <https://doi.org:DOI> 10.1021/ja960510m

15 Kroenke, C. D., Loria, J. P., Lee, L. K., Rance, M. & Palmer, A. G. Longitudinal and transverse 1H-15N/ dipolar15 N chemical shift anisotropy relaxation interference:: Unambiguous determination of rotational diffusion tensors and chemical exchange effects in biological macromolecules. *J Am Chem Soc* **120**, 7905-7915 (1998). <https://doi.org:DOI> 10.1021/ja980832l

16 Yang, D. W. Probing Protein Side Chain Dynamics Via C-13 NMR Relaxation. *Protein Peptide Lett* **18**, 380-395 (2011). <https://doi.org:Doi> 10.2174/092986611794653932

17 Zhang, X., Sui, X. G. & Yang, D. W. Probing methyl dynamics from C-13 autocorrelated and cross-correlated relaxation. *J Am Chem Soc* **128**, 5073-5081 (2006). <https://doi.org:10.1021/ja057579r>

18 Lesovoy, D. M. *et al.* NMR relaxation parameters of methyl groups as a tool to map the interfaces of helix-helix interactions in membrane proteins. *Journal of Biomolecular Nmr* **69**, 165-179 (2017). <https://doi.org:10.1007/s10858-017-0146-1>

19 Agback, T. *et al.* Combined NMR and molecular dynamics conformational filter identifies unambiguously dynamic ensembles of Dengue protease NS2B/NS3pro. *Commun Biol* **6** (2023). <https://doi.org:10.1038/s42003-023-05584-6>

20 Eastman, P. *et al.* OpenMM 7: Rapid development of high performance algorithms for molecular dynamics. *Plos Comput Biol* **13**, e1005659 (2017). <https://doi.org:10.1371/journal.pcbi.1005659>

21 Schwalbe, H. *et al.* The future of integrated structural biology. *Structure* **32**, 1563-1580 (2024). <https://doi.org:10.1016/j.str.2024.08.014>

22 Leontyev, I. & Stuchebrukhov, A. Accounting for electronic polarization in non-polarizable force fields. *Physical Chemistry Chemical Physics* **13**, 2613-2626 (2011). <https://doi.org:10.1039/c0cp01971b>

23 Zeron, I. M., Abascal, J. L. F. & Vega, C. A force field of Li, Na, K, Mg, Ca, Cl, and SO42-in aqueous solution based on the TIP4P/2005 water model and scaled charges for the ions. *Journal of Chemical Physics* **151** (2019). <https://doi.org:Artn> 13450410.1063/1.5121392

24 Kirby, B. J. & Jungwirth, P. Charge Scaling Manifesto: A Way of Reconciling the Inherently Macroscopic and Microscopic Natures of Molecular Simulations. *Journal of Physical Chemistry Letters* **10**, 7531-7536 (2019). <https://doi.org:10.1021/acs.jpclett.9b02652>

25 Nencini, R. *et al.* Effective Inclusion of Electronic Polarization Improves the Description of Electrostatic Interactions: The prosECCo75 Biomolecular Force Field. *Journal of Chemical Theory and Computation* **20**, 7546-7559 (2024). <https://doi.org:10.1021/acs.jctc.4c00743>

26 Onufriev, A. V. & Izadi, S. Water models for biomolecular simulations. *Wires Comput Mol Sci* **8** (2018). <https://doi.org:ARTN> e1347 10.1002/wcms.1347

27 Shi, R., Cooper, A. J. & Tanaka, H. Impact of hierarchical water dipole orderings on the dynamics of aqueous salt solutions. *Nat Commun* **14**, 1-10 (2023). <https://doi.org:ARTN> 4616 10.1038/s41467-023-40278-x

28 Abraham, M. J. *et al.* GROMACS: High performance molecular simulations through multi-level parallelism from laptops to supercomputers. *SoftwareX* **1-2** (2015).

29 Best, R. B. *et al.* Optimization of the Additive CHARMM All-Atom Protein Force Field Targeting Improved Sampling of the Backbone φ, ψ and Side-Chain χ and χ Dihedral Angles. *Journal of Chemical Theory and Computation* **8**, 3257-3273 (2012). <https://doi.org:10.1021/ct300400x>

30 Schwerdtfeger, P. & Wales, D. J. 100 Years of the Lennard-Jones Potential. *Journal of Chemical Theory and Computation* **20**, 3379-3405 (2024). <https://doi.org:10.1021/acs.jctc.4c00135>

31 Qiu, Y. J., Shan, W. J. & Zhang, H. Y. Force Field Benchmark of Amino Acids. 3. Hydration with Scaled Lennard-Jones Interactions. *J Chem Inf Model* **61**, 3571-3582 (2021). <https://doi.org:10.1021/acs.jcim.1c00339>

32 Yoo, J. & Aksimentiev, A. New tricks for old dogs: improving the accuracy of biomolecular force fields by pair-specific corrections to non-bonded interactions. *Phys Chem Chem Phys* **20**, 8432-8449 (2018). <https://doi.org:10.1039/C7CP08185E>

33 Vanommeslaeghe, K., Raman, E. P. & MacKerell, A. Automation of the CHARMM General Force Field (CGenFF) II: Assignment of Bonded Parameters and Partial Atomic Charges. *J Chem Inf Model* **52**, 3155-3168 (2012). <https://doi.org:10.1021/ci3003649>

34 Vanommeslaeghe, K. & MacKerell, A. D. Automation of the CHARMM General Force Field (CGenFF) I: Bond Perception and Atom Typing. *J Chem Inf Model* **52**, 3144-3154 (2012). <https://doi.org:10.1021/ci300363c>

35 Shi, Y. *et al.* Polarizable Atomic Multipole-Based AMOEBA Force Field for Proteins. *Journal of Chemical Theory and Computation* **9**, 4046-4063 (2013). <https://doi.org:10.1021/ct4003702>

36 Wang, A. H., Zhang, Z. C. & Li, G. H. Higher Accuracy Achieved in the Simulations of Protein Structure Refinement, Protein Folding, and Intrinsically Disordered Proteins Using Polarizable Force Fields. *Journal of Physical Chemistry Letters* **9**, 7110-7116 (2018). <https://doi.org:10.1021/acs.jpclett.8b03471>

37 Cohen, F. E. & Sternberg, M. J. E. On the Prediction of Protein-Structure - the Significance of the Root-Mean-Square Deviation. *J Mol Biol* **138**, 321-333 (1980). <https://doi.org:Doi> 10.1016/0022-2836(80)90289-2

38 Fushman, D., Tjandra, N. & Cowburn, D. Direct measurement of 15N chemical shift anisotropy in solution. *J Am Chem Soc* **120**, 10947-10952 (1998). <https://doi.org:DOI> 10.1021/ja981686m

39 Robson, S. A., Dag, Ç., Wu, H. W. & Ziarek, J. J. TRACT revisited: an algebraic solution for determining overall rotational correlation times from cross-correlated relaxation rates. *Journal of Biomolecular Nmr* **75**, 293-302 (2021). <https://doi.org:10.1007/s10858-021-00379-5>

40 Yao, L. S., Vögeli, B., Ying, J. F. & Bax, A. NMR Determination of Amide N-H Equilibrium Bond Length from Concerted Dipolar Coupling Measurements. *J Am Chem Soc* **130**, 16518-+ (2008). <https://doi.org:10.1021/ja805654f>

41 Chill, J. H., Louis, J. M., Baber, J. L. & Bax, A. Measurement of N relaxation in the detergent-solubilized tetrameric KcsA potassium channel. *Journal of Biomolecular Nmr* **36**, 123-136 (2006). <https://doi.org:10.1007/s10858-006-9071-4>

42 Willmott, C. J. & Matsuura, K. Advantages of the mean absolute error (MAE) over the root mean square error (RMSE) in assessing average model performance. *Climate Res* **30**, 79-82 (2005). <https://doi.org:DOI> 10.3354/cr030079

43 Homayouni, R., Heinrich, K., Wei, L. & Berry, M. W. Gene clustering by latent semantic indexing of MEDLINE abstracts. *Bioinformatics* **21**, 104-115 (2005). <https://doi.org:10.1093/bioinformatics/bth464>
